# HIF factors cooperate with PML-RARα to promote acute promyelocytic leukemia progression and relapse

**DOI:** 10.1002/emmm.201303065

**Published:** 2014-04-07

**Authors:** Nadia Coltella, Stefano Percio, Roberta Valsecchi, Roberto Cuttano, Jlenia Guarnerio, Maurilio Ponzoni, Pier Paolo Pandolfi, Giovanni Melillo, Linda Pattini, Rosa Bernardi

**Affiliations:** 1 Division of Molecular Oncology, Leukemia Unit, San Raffaele Scientific InstituteMilan, Italy; 2 Department of Electronics, Information and Bioengineering, Politecnico di MilanoMilan, Italy; 3 Pathology Unit, Leukemia Unit, San Raffaele Scientific InstituteMilan, Italy; 4 Department of Medicine and Pathology, Cancer Research Institute, Beth Israel Deaconess Cancer Center, Beth Israel Deaconess Medical Center, Harvard Medical SchoolBoston, MA, USA; 5 Science Applications International Corporation-Frederick, Inc., Frederick National Laboratory for Cancer ResearchFrederick, MD, USA

**Keywords:** acute promyelocytic leukemia, hypoxia-inducible transcription factor, leukemia-initiating cells, mouse models, PML-RARα

## Abstract

Acute promyelocytic leukemia (APL) is epitomized by the chromosomal translocation t(15;17) and the resulting oncogenic fusion protein PML-RARα. Although acting primarily as a transcriptional repressor, PML-RARα can also exert functions of transcriptional co-activation. Here, we find that PML-RARα stimulates transcription driven by HIF factors, which are critical regulators of adaptive responses to hypoxia and stem cell maintenance. Consistently, HIF-related gene signatures are upregulated in leukemic promyelocytes from APL patients compared to normal promyelocytes. Through *in vitro* and *in vivo* studies, we find that PML-RARα exploits a number of HIF-1α-regulated pro-leukemogenic functions that include cell migration, bone marrow (BM) neo-angiogenesis and self-renewal of APL blasts. Furthermore, HIF-1α levels increase upon treatment of APL cells with all-*trans* retinoic acid (ATRA). As a consequence, inhibiting HIF-1α in APL mouse models delays leukemia progression and exquisitely synergizes with ATRA to eliminate leukemia-initiating cells (LICs).

## Introduction

Acute promyelocytic leukemia (APL) is a sub-type of acute myeloid leukemia (AML) distinguished by a differentiation block at the promyelocytic stage and the largely predominant t(15;17) chromosomal translocation generating the PML-RARα fusion protein (Ablain & de The, 2011). PML-RARα acts mainly as a transcriptional repressor, by blocking expression of a number of genes involved in myeloid differentiation through relaxed DNA-binding capacity and recruitment of transcriptional co-repressors and chromatin modifiers (Ablain & de The, 2011). Nonetheless, PML-RARα also exerts functions of transcriptional co-activation toward transcription factors like AP-1 and GATA-2 (Doucas *et al*, 1993; Tsuzuki *et al*, 2000), and induces expression of genes promoting stem cells maintenance (Viale *et al*, 2009). Moreover, treatment of APL cells with therapeutic doses of all-*trans* retinoic acid (ATRA) induces transcriptional activation by PML-RARα, followed by PML-RARα degradation and rapid and effective APL blast differentiation and leukemia de-bulking (Ablain & de The, 2011).

Hypoxia-inducible transcription factors (HIFs) are often upregulated in solid tumors and foster tumorigenesis in several ways, which include cell migration, neo-angiogenesis and maintenance of cancer stem cells (Lu & Kang, 2010; Lee & Simon, 2012). In lymphoma and AML, HIF-1α was recently found upregulated specifically in LICs, where it was shown to stimulate self-renewal (Lee & Simon, 2012). Using a combination of *in vitro* and *in vivo* studies and *in silico* analysis of patients' gene expression profiles, we show that in APL, PML-RARα functionally cooperates with HIF factors, particularly HIF-1α, and exploits the transcriptional repertoire of HIF-1α to promote not only LICs maintenance but also leukemia progression at multiple levels.

## Results

### PML-RARα is a HIF-α transcriptional co-activator

**Figure 1:**
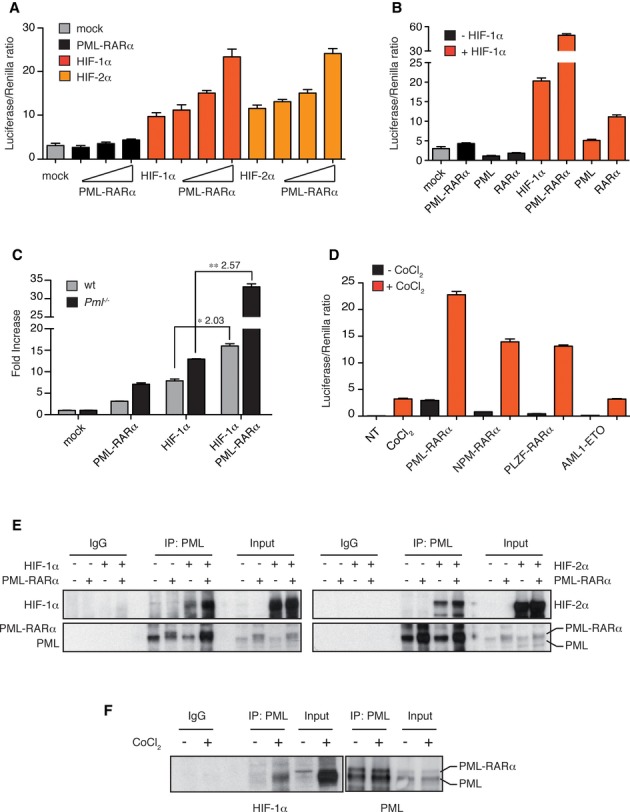
PML-RARa is a HIF-α transcriptional co-activator. A HIF-α transactivation assays (A–D) with HRE-luciferase construct. Results are presented as Luciferase/Renilla ratio (mean ± s.e.m. of experiments performed in triplicate). B HEK-293 cells transfected with stable mutants of HIF-1α or HIF-2α and increasing concentrations of PML-RARα. C HEK-293 cells transfected with a stable form of HIF-1α along with PML-RARα, PML or RARα. D Wild-type and *Pml*^−/−^ MEFs transfected with HIF-1α and PML-RARα. Asterisks indicate fold change induction of HIF-1α-mediated transactivation upon PML-RARα expression. E HEK-293 cells transfected with the indicated fusion genes and treated with CoCl_2_. F Co-immunoprecipitation of exogenous stable forms of HIF-1α (left panel) and HIF-2α (right panel) and PML-RARα with a PML-directed antibody in HEK-293 cells. Of note, exogenously expressed PML-RARα migrates very closely to endogenous PML. Co-immunoprecipitation of endogenous HIF-1α and PML-RARα with a PML-directed antibody in NB4 cells treated with CoCl_2_. Data information: All experiments were repeated at least twice. Source data are available for this figure.

We have previously reported that PML inhibits HIF-1α-mediated transcription (Bernardi *et al*, 2006). Because PML-RARα is a dominant-negative inhibitor of PML (Daniel *et al*, 1993), we asked whether PML-RARα activated HIF-1α through PML inhibition. Transcription assays showed that PML-RARα activated transcription by HIF-1α and HIF-2α in a dose-dependent manner (Fig 1A), although both PML and RARα inhibited HIF-1α-mediated transcription (Fig 1B). However, HIF-1α activation by PML-RARα did not depend on PML inhibition as it also occurred in *Pml*^−/−^ mouse embryonic fibroblasts (MEFs), regardless of higher basal HIF-1α activity (Bernardi *et al*, 2006) (Fig 1C). Also, increased HIF activity was not caused by protein accumulation (Supplementary Fig S1A and B) and was not further stimulated by ATRA treatment (Supplementary Fig S1C), in contrast to PML-RARα-mediated activation of AP-1 and GATA-2 (Doucas *et al*, 1993; Tsuzuki *et al*, 2000). Interestingly, other APL fusion proteins PLZF-RARα and NPM-RARα also activated transcription by HIF-1α upon stabilization by the hypoxia-mimetic agent cobalt chloride (CoCl_2_, which at the conditions used in our assays induced HIF-1α and not HIF-2α transcriptional activity, Supplementary Fig S1D and E), while fusion proteins of other AML sub-types such as AML1-ETO did not (Fig 1D). These data suggest that activation of HIF factors may be a common event in APL.

To assess whether the functional cooperation of PML-RARα and HIF-1α is mediated by physical interaction, co-immunoprecipitation experiments were performed in HEK-293 and NB4 cells. Overexpressed stable forms of HIF-1α and HIF-2α as well as endogenous HIF-1α were co-immunoprecipitated with PML-RARα and with PML (Fig 1E and F); however, they did not directly interact *in vitro* (Supplementary Fig S1F and G).

Taken together, these data indicate that PML-RARα functionally cooperates with HIF factors independently of PML inhibition, HIF-α accumulation, ATRA binding and direct interaction. The molecular mechanisms driving PML-RARα-HIF cooperation remain to be elucidated, but may involve indirect interactions within larger protein complexes. Also, these interactions could be mediated by both moieties of PML-RARα, because both PML and RARα interfere with HIF-mediated transcription, PML interacts with HIF-α, and different RARα fusion proteins activate HIF-1α. This suggests that PML-RARα may hijack physiological functions of PML and RARα toward a new functional interaction with HIF-1α.

### HIF-regulated gene signatures are upregulated in human APL

**Figure 2:**
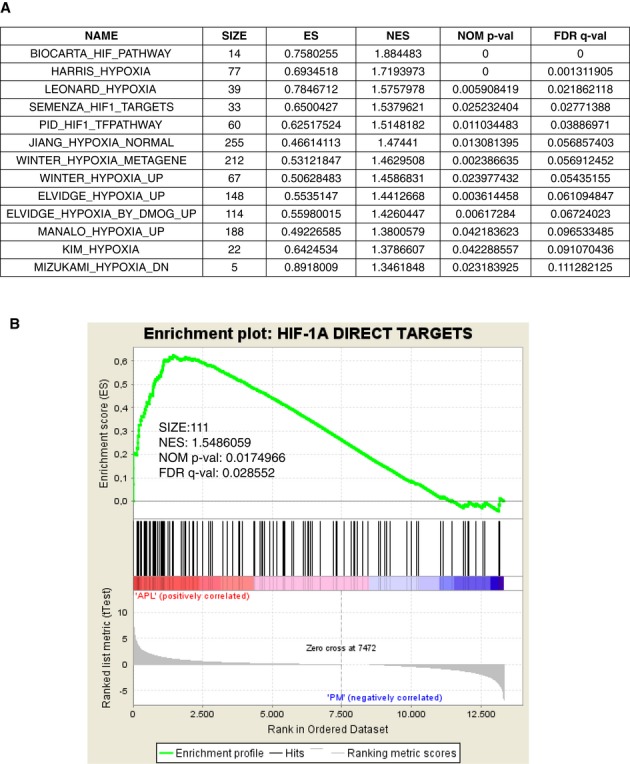
HIF-1α-dependent gene signatures are overexpressed in human APL promyelocytes compared to normal promyelocytes. A MSigDb signatures related to hypoxia and HIF-1α pathways enriched in the comparison between APL and normal promyelocytes gene expression. Enrichment scores (ES) and normalized ES (NES) accounting for the gene set size are reported. Significance was estimated in terms of nominal *P*-value and FDR *Q*-value. B GSEA plot for the comparison between APL and normal promyelocytes (PM) gene expression showing significant enrichment of HIF-1α direct targets in the APL-upregulated genes.

To understand whether the functional cooperation of PML-RARα with HIF-1α is relevant to APL pathogenesis, expression of hypoxia-regulated gene signatures was evaluated in microarray data of normal and leukemic promyelocytes from APL patients (Marstrand *et al*, 2010). A number of HIF-related gene signatures were found significantly correlated with the distinction between leukemic promyelocytes and normal human promyelocytes (Fig 2A). To gain a better understanding of the direct role of HIF-1α in human APL, a list of *bona fide* HIF-1α target genes was constructed based on current literature (Wenger *et al*, 2005) by selecting HIF-1α targets validated in multiple experimental settings and provided with functional HIF-1α binding sites (Supplementary Table S1). Enrichment analysis revealed that genes belonging to this signature were significantly enriched in the genes upregulated in APL vs normal promyelocytes (Fig 2B), thus confirming that direct transcriptional targets of HIF-1α are upregulated in APL and that activation of HIF-regulated signatures is relevant for human APL.

### HIF-1α inhibition impairs APL cell migration, neo-angiogenesis and self-renewal

**Figure 3:**
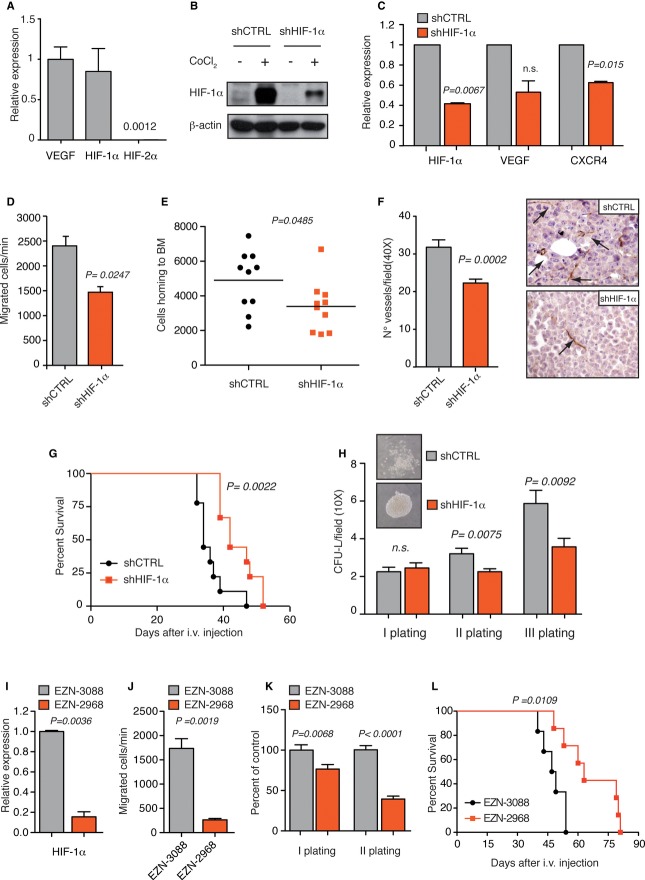
HIF-1α inhibition impairs APL progression in a xenograft model. A Real-time PCR analysis of VEGF, HIF-1α and HIF-2α (the number indicates HIF-2α expression relative to VEGF) in NB4 cells. B Immunoblot of HIF-1α in NB4 cells transduced with lentiviral vectors carrying control (shCTRL) or HIF-1α-directed shRNA (shHIF-1α) and treated with CoCl_2_. C Real-time PCR analysis of HIF-1α, VEGF and CXCR4 in shHIF-1α NB4 cells relative to shCTRL cells. D Spontaneous migration of shCTRL or shHIF-1α NB4 cells. Data are expressed as mean values ± s.e.m of triplicates from one representative experiment out of three with similar results. E Total numbers of human CD13^+^ shCTRL or shHIF-1α NB4 cells localizing to the BM of *Rag2*^−/−^γ*c*^−/−^ mice 16 h after tail vein injection (over 2.5 × 10^6^ events; *n* = 10, from two independent experiments). F CD31 immunostaining of BM colonized by shCTRL or shHIF-1α NB4 cells. Graph on the left shows average number of microvessels per field (40×). Pictures on the right show representative images (arrows indicate microvessels). G Kaplan–Meier survival curve of *Rag2*^−/−^γ*c*^−/−^ mice injected with shCTRL or shHIF-1α NB4 cells (*n* = 9). H Colony-forming assay upon serial re-plating of shCTRL or shHIF-1α NB4 cells. Data represent the number of colonies per field (10×). Upper panels: Representative pictures of colonies formed by shCTRL or shHIF-1α NB4 cells in methylcellulose. I Real-time PCR analysis of HIF-1α in NB4 cells 24 h after transfection with EZN-2968 relative to cells transfected with EZN-3088. J Spontaneous migration of NB4 cells 24 h after transfection with EZN-3088 or EZN-2968. K Colony-forming assay of EZN-3088 or EZN-2968-transfected NB4 cells. For the first plating, cells were seeded 24 h after transfection. L Kaplan–Meier survival curve of *Rag2*^−/−^γ*c*^−/−^ mice injected with EZN-3088 and EZN-2968-transfected NB4 cells 24 h after transfection (*n* = 6 for EZN-3088 group and *n* = 7 for EZN-2968 group). Data information: Results from migration and colony-forming assays are expressed as mean values ± s.e.m of triplicates from one representative experiment. All experiments were repeated at least twice. Source data are available for this figure.

To study the functions of endogenous HIF factors in APL, HIF-1α and HIF-2α expressions were analyzed in APL NB4 cells. HIF-1α mRNA was significantly expressed, similarly to VEGF, which is highly expressed in APL patients (Gutierrez *et al*, 2005), while HIF-2α levels were very low (Fig 3A). Consistently, HIF-1α was detected by Western blotting upon CoCl_2_ treatment but also in normoxic conditions (Fig 3B). The mechanisms leading to normoxic HIF-1α expression in NB4 cells are unknown, but may not depend on PML-RARα as PML-RARα overexpression did not modulate HIF-1α protein (Supplementary Fig S1A and B). Because it was recently reported that HIF-1α is regulated in an oxygen-independent manner in hematopoietic stem cells (Nombela-Arrieta *et al*, 2013), it is possible that in some hematopoietic cells oxygen-sensing mechanisms are tuned down to allow basal expression of HIF factors.

Chronic HIF-1α down-regulation by shRNA led to specific inhibition of HIF-1α and down-regulation of *bona fide* HIF target genes in NB4 cells (Fig 3B and C and Supplementary Fig S2A). To understand the role of HIF-1α in APL, we analyzed a number of known HIF-regulated functions: in NB4 cells, HIF-1α down-regulation did not affect proliferation (Supplementary Fig S2B), but impaired basal cell migration (Fig 3D) and *in vivo* BM homing (Fig 3E), consistently with CXCR4 down-regulation (Fig 3C). Conversely, *in vitro* migration toward SDF-1 or VEGF did not occur (Supplementary Fig S2C) due to receptor internalization in culture (Tavor *et al*, 2004). Next, as APL patients have increased BM neo-angiogenesis and high VEGF levels (Kini *et al*, 2001), and VEGF expression in regulated by HIF-1α in NB4 (Fig 3C), microvessel density was analyzed *in vivo*. HIF-1α down-regulation significantly inhibited neo-angiogenesis both in BM (Fig 3F) as well as in other tumor masses (Supplementary Fig S2D). Also, HIF-1α down-regulation significantly increased the survival of immunocompromised mice (Fig 3G).

Finally, as it was recently reported that HIF-1α regulates maintenance of LICs (Wang *et al*, 2011), we analyzed the effect of HIF-1α inhibition on leukemia self-renewal *in vitro*. Methylcellulose re-plating assays showed that NB4 CFU-L (colony-forming unit leukemia) increased upon serial re-plating (Lin *et al*, 2010) only in cells expressing HIF-1α (Fig 3H), thus indicating that self-renewal of CFU-L requires HIF-1α. Also, NB4 colonies appeared more compact upon HIF-1α silencing, consistently with decreased cell migration (Fig 3H).

Inhibition of HIF-1α with an unrelated shRNA confirmed the impairment in cell migration and CFU-L re-plating efficacy and prolonged mice survival in xenograft experiments (Supplementary Fig S3A–E). Unexpectedly, stable silencing of HIF-2α also impaired NB4 CFU-L re-plating efficacy and slightly prolonged mice survival, although not affecting cell migration (Supplementary Fig S3B–E). This occurred despite the low expression of HIF-2α in NB4 cells, which was also observed in APL patients (Fig 3A and Supplementary Fig S3F). Interestingly, it was recently shown that HIF-2α promotes maintenance of human CD34^+^ hematopoietic stem cells (Rouault-Pierre *et al*, 2013), although we found that also in this context HIF-2α is expressed at lower levels than HIF-1α (Supplementary Fig S3G). Therefore, it appears that HIF-2α exerts critical functions in normal and transformed hematopoietic cells regardless of its expression levels.

Based on the results obtained upon HIF-1α chronic silencing, we next wished to test the consequences of acute HIF-1α inhibition. The RNA antagonist EZN-2968 is a locked nucleic acid-modified oligonucleotide (LNA-ON) specifically targeting HIF-1α, while EZN-3088 is a control LNA-ON (Greenberger *et al*, 2008). Strong inhibition of HIF-1α by EZN-2968 (Fig 3I) recapitulated more efficiently what was previously observed by chronic HIF-1α silencing: blockade of cell migration and CFU-L efficacy upon re-plating (Fig 3J and K), as well as delayed leukemia progression (Fig 3L).

Finally, to substantiate our experiments in another APL cell context, we took advantage of the U937-PR9 cell line where PML-RARα is expressed under the control of a zinc (Zn)-inducible promoter (Grignani *et al*, 1998). Interestingly, acute induction of PML-RARα with Zn increased HIF-1α, GLUT1 and CXCR4 mRNA levels, while having no significant effect on U937-MT control cells (Supplementary Fig S4A and B). Strong inhibition of HIF-1α in U937-PR9 cells impaired cell migration and colony formation more efficiently when PML-RARα was expressed, albeit also affecting migration and clonogenicity in the absence of PML-RARα (Supplementary Fig S4C–E). These data indicate that HIF-1α may play a role also in leukemic contexts different from APL, but forced expression of PML-RARα further sensitizes leukemic cells to its inhibition.

Taken together, our data indicate that hampering HIF-1α functions affects different aspects of APL biology that include cell migration and self-renewal, thus resulting in delayed leukemia progression.

### HIF-1α inhibition synergizes with ATRA treatment to eradicate APL

**Figure 4:**
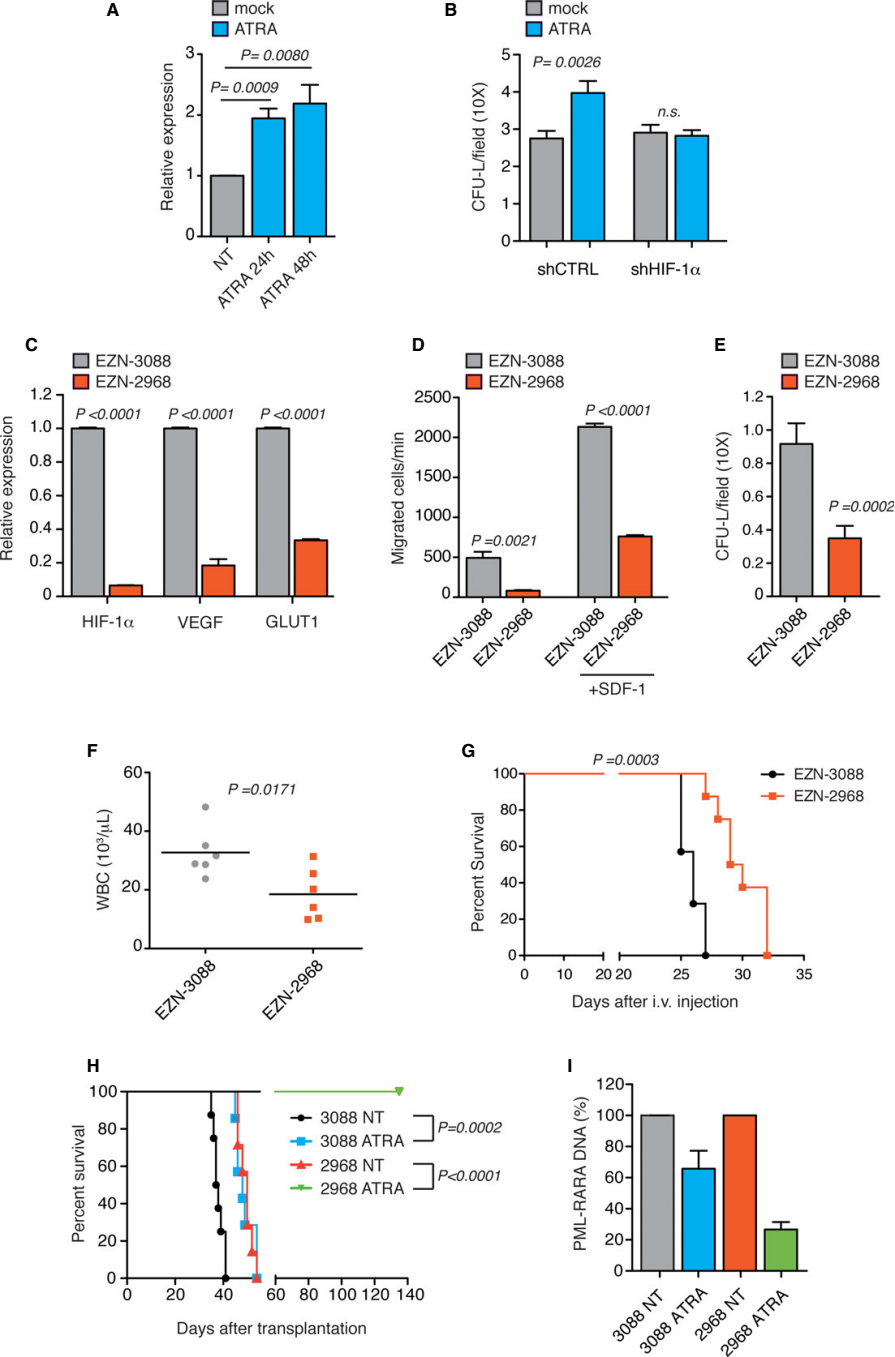
HIF-1α inhibition synergizes with ATRA toward LICs eradication. A Real-time PCR analysis of HIF-1α in NB4 cells treated with 1 μM ATRA for the indicated times relative to untreated cells. B Colony-forming assay of NB4 shCTRL or shHIF-1α cells pre-treated for 24 h with 1 μM ATRA and plated in methylcellulose after drug washout. Data represent number of colonies per field (10×). C Real-time PCR analysis of HIF-1α, VEGF and GLUT1 in mouse leukemic cells 24 h after transfection with EZN-2968 relative to cells transfected with EZN-3088. D Spontaneous and SDF-1-induced migration of mouse leukemic cells 24 h after transfection with EZN-3088 or EZN-2968. E Colony-forming assay of mouse leukemic cells 24 h after transfection with EZN-3088 or EZN-2968. F Peripheral white blood cell counts of 129Sv mice injected with mouse APL blasts 24 h after transfection with EZN-3088 or EZN-2968. Blood was collected at day 21 (*n* = 6). G Kaplan–Meier survival curve of 129Sv mice injected with mouse APL blasts 24 h after transfection with the corresponding LNA-ON (*n* = 7 for EZN-3088 and *n* = 8 for EZN-2968). H Kaplan–Meier survival curve of secondary recipient mice injected with BM cells from donor mice injected with APL blasts transfected with EZN-3088 or EZN-2968 and treated for 4 days with ATRA at day 21 post-leukemia challenge (*n* = 8 for EZN-3088 and EZN-2968 ATRA;*n* = 7 for EZN-3088 ATRA and EZN-2968). I PML-RARα DNA real-time PCR analysis of BM cells extracted from donor leukemic mice injected with APL blasts transfected with EZN-3088 or EZN-2968 and treated for 4 days with ATRA at day 21 post-leukemia challenge (*n* = 3). Data are presented as percentage of ATRA-treated over reciprocal untreated (NT) mice.

It has long been known that ATRA treatment promotes rapid APL blast differentiation but is not sufficient to eradicate LICs and rarely leads to prolonged disease remission (Sanz *et al*, 2008; Lo-Coco *et al*, 2010). Additionally, *in vitro* studies suggest that ATRA may promote the expansion of APL LICs (Zheng *et al*, 2007). Interestingly, we found that ATRA treatment induced HIF-1α expression (Fig 4A) and increased clonogenicity in a HIF-1α-dependent manner in NB4 cells (Fig 4B).

To understand whether HIF-1α inhibition led to LICs elimination especially upon ATRA treatment, we turned to a mouse APL model (Minucci *et al*, 2002). Lineage-negative BM cells were transduced with PML-RARα followed by transplantation into lethally irradiated syngeneic mice. Transplanted mice developed a fatal myeloid leukemia that recapitulates human APL with peripheral leukocytosis, colonization of the BM and spleen and responsiveness to ATRA (Minucci *et al*, 2002; Fig 4). We first aimed at confirming the effects of HIF-1α inhibition in mouse APL. *Ex vivo* APL blasts electroporation with EZN-2968 led to significant impairment in the expression of HIF-1α and HIF-target genes (Fig 4C) and inhibited basal and SDF-1-directed cell migration, CFU-L formation and leukemia involvement in peripheral blood (Fig 4D-F), leading to increased mice survival (Fig 4G).

To next assess whether HIF-1α inhibition cooperated with ATRA to eliminate LICs *in vivo*, APL cells were transfected with EZN-2968 or EZN-3088 and transplanted into syngeneic mice. Leukemic mice were treated with ATRA for 4 days, after which BM was transplanted into recipient mice to measure leukemia engraftment by LICs. Strikingly, while short ATRA treatment or HIF-1α silencing only modestly affected the survival of transplanted animals, combined treatment exquisitely synergized in preventing leukemia engraftment (Fig 4H). Moreover, HIF-1α inhibition also synergized with ATRA in promoting leukemia de-bulking (Fig 4I), therefore suggesting that a strong inhibition of HIF-1α may cooperate with ATRA not only to blunt the leukemia-initiating capacity of APL cells, but also their *in vivo* survival and/or differentiation.

## Discussion

PML-RARα is a gain of function transcription factor that is believed to exert leukemogenic functions mostly by acting as a transcriptional repressor. Here, we define a new pro-leukemogenic axis exploited by PML-RARα through its synergistic cooperation with HIF transcription factors. We find that despite both PML and RARα inhibit HIF-1α-mediated transcription, the fusion protein PML-RARα acts as a HIF-α transcriptional co-activator. Accordingly, HIF-dependent signatures are upregulated in leukemic blasts of APL patients, and binding sites for the HIF-α heterodimerization partner HIF-1β are enriched in the promoters of genes preferentially expressed in APL blasts (Marstrand *et al*, 2010), thus indicating that activation of HIF factors is relevant to the pathophysiology of APL.

Our findings challenge the current view that PML-RARα exerts oncogenic functions by transcriptional repression, while switching to a transcriptional activator upon ATRA treatment, and indicate that important functions of PML-RARα may also be mediated by transcriptional co-activation. The detailed molecular mechanisms by which PML-RARα co-activates HIF factors are still unknown. Because PML-RARα indirectly interacts with HIF-α in cells and binds to the promoters of *bona fide* HIF target genes (Martens *et al*, 2010; Wang *et al*, 2010), it is possible that within specific DNA domains rather than binding transcriptional co-repressors, PML-RARα partakes to activating complexes that facilitate HIF-mediated transcription. Moreover, as acute induction of PML-RARα increased HIF-1α mRNA, it is also possible that in patients, HIF-1α is upregulated upon PML-RARα expression, therefore adding an additional level of cooperation between PML-RARα and HIF-1α.

The cooperation of PML-RARα with HIF-1α, and to a minor extent HIF-2α, is relevant to APL pathogenesis by impacting on a number of HIF-mediated functions, including spontaneous and chemokine-dependent cell migration, and tumor neo-angiogenesis. In addition, HIF-1α regulates self-renewal of APL leukemic blasts, which is coherent with the role of HIF factors in regulating stem cell maintenance in other hematopoietic contexts (Takubo *et al*, 2010; Wang *et al*, 2011; Zhang *et al*, 2012; Rouault-Pierre *et al*, 2013). However, in contrast to other hematological malignancies where HIF-1α is found expressed predominantly in LICs (Wang *et al*, 2011; Zhang *et al*, 2012), in APL, HIF-1α activation is triggered by a cooperative event with PML-RARα, whose expression is not confined to LICs. Indeed, proof-of-principle experiments show that inhibition of HIF-1α delays APL progression and prolong survival of leukemic mice.

More remarkably, HIF-1α is also involved in regulating LICs maintenance upon ATRA treatment. Specifically, ATRA increases expression of HIF-1α in APL and other cell types (Meani *et al*, 2005), and HIF-1α inhibition exquisitely cooperates with ATRA toward reducing APL LICs. In conclusion, our studies demonstrate that HIF factors are important regulators of APL pathogenesis and response to therapy.

## Materials and Methods

### Cell culture and reagents

NB4, U937-PR9 and U937-MT cells (Grignani *et al*, 1998) (kindly provided by S. Minucci) and mouse APL cells were maintained in RPMI 1640; HEK-293 and Phoenix-ECO in DMEM and HEK-293T in IMDM media supplemented with 10% FBS and antibiotics (Lonza). Primary mouse embryonic fibroblasts (MEFs) from wild-type and *Pml*^−/−^ embryos were prepared as previously described (Bernardi *et al*, 2004). All cell lines were maintained at 37°C in humidified atmosphere containing 5% CO_2_.

EZN-3088 (control LNA-ON for HIF-1α) and EZN-2968 (LNA-ON for HIF-1α) (Greenberger *et al*, 2008) were provided by Belrose Pharma Inc. and used in accordance with the manufacturer's instructions; ATRA, CoCl_2_ and Zn were purchased from Sigma; ATRA pellets were from Innovative Research of America. All cytokines were from Peprotech.

### Lentiviral vectors

GIPZ HIF-1α and HIF-2α shRNA or control shRNA plasmids were from Open Biosystems. Lentiviral vectors were obtained by HEK-293T transfection with calcium phosphate and subsequent concentration as previously described (Follenzi *et al*, 2000). HEK-293 and NB4 cells were transduced by spinoculation and sorted for GFP expression at least 2 weeks post-infection.

### Immunoprecipitation and immunoblot

When indicated, NB4 cells (shCTRL or shHIFs) were treated for 24 h with 100 μM CoCl_2_ before lysis. For Western blot, proteins were extracted with RIPA buffer (Sigma) supplemented with protease inhibitor cocktail (Roche); for luciferase assays, cells were lyzed in passive lysis buffer (Promega); for co-immunoprecipitation experiments, cells were lyzed after mild cross-linking with 0.4% formaldehyde (7 min at RT) in CO-IP buffer (10 mM NaCl, 10 mM Tris–HCl pH 7.5, 5 mM MgCl_2_, 0.5% CHAPS) supplemented with protease and phosphatase inhibitors (Pierce) and then briefly sonicated to extract nuclear proteins. Total lysates were immunoprecipitated with an antibody against PML (PG-M3; Santa Cruz).

Cell extracts and immunoprecipitates were resolved by SDS–PAGE 7.5–10% and transferred to a PVDF membrane (Biorad). Non-specific binding was blocked in 5% non-fat milk for 1 h at RT and blotted with the following antibodies: rabbit polyclonal HIF-1α (Cayman), rabbit polyclonal PML (Santa Cruz) and rabbit polyclonal PML (Novus). Mouse β-actin (Sigma) was used as internal loading control.

### *In vitro* binding assays

Histidine-tagged human HIF-1α full-length protein (His-HIF-1α) was purchased from Abcam. GST-tagged HIF-2α full-length protein (GST-HIF-2α) was purchased from Abnova. Biotin-labeled PML-RARα protein was generated in reticulocyte lysates using the TNT T7 coupled transcription/translation system (Promega). 30 μL of *in vitro*-translated biotin-labeled PML-RARα was mixed with 5 μg of His or His-HIF-1α, GST or GST-HIF-2α in a final volume of 1 mL of binding buffer (10 mM NaCl, 10 mM Tris–HCl pH 7.5, 5 mM MgCl_2_, 0.5% CHAPS and 5 mM imidazole for HIF-1α) and incubated O/N at 4°C followed by the addition of 40 μl of HIS-Select^**®**^ Nichel Affinity Gel (Sigma) or glutathione–Sepharose 4B beads (GE Healthcare) and a further incubation at 4°C for 2 h. After washing, beads were analyzed by SDS–PAGE. His and GST proteins were kindly provided by V. M. Neguembor.

The following antibodies were used: Streptavidin-HRP (Sigma), mouse monoclonal GST (Sigma) and mouse monoclonal HIF-1α (BD Biosciences).

### Luciferase assays

Cells were plated in 24-well plates (Costar) and transfected by Lipofectamine 2000 (Invitrogen) with a reporter plasmid expressing the luciferase gene under the control of a hypoxia responsive element (HRE)-containing promoter (kind gift of C. Simon). Plasmids containing PML, RARα, PML-RARα, NPM-RARα, PLZF-RARα and AML1-ETO or plasmids containing stable forms of HIF-1α and HIF-2α, or wild-type HIF-1α for experiments in MEFs (kind gifts of C. Simon) were transfected where indicated. HEK-293 cells were treated for 8 h with 300 μM CoCl_2_. Renilla-expressing plasmid was co-transfected for transfection normalization. Dual-Luciferase® Reporter Assay System (Promega) was used to measure firefly and Renilla luciferase activities and their ratio calculated in a GloMax® luminometer (Promega).

### Real-time PCR

RNA was isolated with the RNeasy mini kit (Qiagen). cDNA was obtained by retro-transcription of 1 μg total RNA using Advantage RT for PCR kit (Clontech) and analyzed by real-time PCR in 7900 Fast Real-Time PCR System (Applied Biosystem).

All probes for TaqMan assays were purchased from Applied Biosystem. 18S was used as internal control. The relative expression of different cDNAs was calculated using the 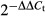 method with respect to control conditions, except for the analysis of VEGF and HIF factors in NB4 cells where data were expressed using the 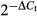 method relative to 18S expression. All data represent mean values of at least two independent experiments.

For PML-RARα DNA real-time PCR, DNA was extracted using QIAamp DNA Micro kit (Qiagen) and quantified as previously described (Nasr *et al*, 2008). 18S was used as internal control.

### Methylcellulose colony-forming assays

For human cell lines, 5 × 10^3^ cells were resuspended in Human Methylcellulose Base Media (R&D) and plated in 35-mm culture dishes. Cells were allowed to grow for 7–10 days and then colonies were pooled, counted, and 5 × 10^3^ cells were re-seeded for re-plating experiments. When indicated Zn (100 μM) was added to the methylcellulose medium. In pre-treatment experiments (Fig 4B), NB4 cells were treated in liquid medium with ATRA (1 μM) for 24 h and plated in methylcellulose after drugs washout. In CFU-L experiments of BM cells from leukemic mice, 3 × 10^4^ cells were seeded in MethoCult® GF Medium (STEMCELL Technologies) 24 h after transfection with EZN-3088 and EZN-2968, and colonies were counted 7–10 days after plating.

### Migration assays

For human cell lines, 1 × 10^6^ cells were seeded in the upper chamber of a transwell 6.5-mm diameter and 5-μM pore (Costar). Cells in the lower chamber were counted by flow cytometer as number of cells acquired per minute (LSR II Beckton Dickinson) 16 h after seeding for NB4 cells and 2.5 h after seeding for U937-PR9 and U937-MT cells. For migration experiments of BM leukemic cells 1.25 × 10^5^ mouse leukemia cells were seeded 24 h after electroporation and migration was assessed after 4 h.

### Animal models

*Rag2*^−/−^γ*c*^−/−^ immunocompromised mice were maintained in pathogen-free animal facility and treated in accordance with European Union guidelines. All animal protocols were approved by the Institutional Animal Care and Use Commitee (IACUC).

For homing experiments, mice were injected intravenously (i.v.) with 5 × 10^6^ NB4 cells (shCTRL or shHIF-1α) and euthanized after 16 h. BM samples were stained with anti-human CD13 antibody. For survival experiments, mice were challenged i.v. with 7 × 10^5^ NB4 cells (shCTRL or shHIFs) and sacrificed when terminally sick.

Transduction of Lin- BM cells from 129Sv mice with a modified retroviral vector (pBABE backbone with ΔNGFR as a reporter) expressing PML-RARα was performed as previously described (Minucci *et al*, 2002). ΔNGFR-sorted cells (1 × 10^6^) were inoculated i.v. into lethally irradiated syngeneic mice. Mice were monitored periodically for clinical signs of disease and leukocytosis. When terminally sick, animals were sacrificed and 1 × 10^6^ leukemic BM cells were inoculated into syngeneic recipients.

Mouse APL cells were transfected *ex vivo* with EZN-2968 and EZN-3088 in an Amaxa™ 4D-Nucleofector™ System (Lonza) and transplanted into syngeneic mice 24 h after transfection. 1 × 10^6^ live cells per recipient mouse were transplanted for the experiments in Fig 4F and G. For the experiments of Fig 4H, 5 × 10^5^ cells transfected with EZN-2968 or EZN-3088 were transplanted in 4 mice/cohort, and mice were treated with ATRA for 4 consecutive days at day 21 after leukemia challenge, when control animals showed leukemia involvement in BM. Second transplantation was performed with 1 × 10^6^ cells. ATRA was administered by subcutaneous implantation of 2 release pellets (5 mg each).

### Statistics

Two-sided *t*-tests were used to validate the significance of the data analyzed and considered statistically significant when *P *<* *0.05. For survival experiments, Kaplan–Meier curves were analyzed with the Mantel–Cox test.

### mRNA expression data

We used the publicly available datasets GSE1729 (Gutierrez *et al*, 2005), GSE1159 (Valk *et al*, 2004) and GSE19556 (Theilgaard-Monch *et al*, 2005) from the NIH Gene Expression Omnibus repository to select mRNA expression data of APL BM samples (*n* = 25) and normal promyelocytes (*n* = 3). The experimental platform was Affymetrix GeneChip Human Genome HG-U133A for all the samples. Raw data (retrieved as * CEL files) were pre-processed using the GCRMA library (Wu & Irizarry, 2004) of the Bioconductor project in the R environment.

To compare the expression levels of HIF-1α and HIF-2α (EPAS1), we retrieved CD34^+^ (*n* = 5) and APL (*n* = 14) samples from the publicly available dataset GSE12662 (Payton *et al* 2009), containing data acquired by means of the Affymetrix HG-U133 Plus 2.0 Array.

### Data mining

Hypoxia related signatures were retrieved from the collection of curated gene sets (c2) of the Molecular Signatures Database (MSigDB). A gene set enrichment analysis (GSEA) (Subramanian *et al*, 2005) was performed either using these publicly available gene sets or applying an *ad hoc* list of direct HIF-1α targets. A *t*-statistic metric was applied to establish the gene ranking, and significance was provided both in terms of nominal *P*-value and as FDR *Q*-value to take into account the adjustment for multiple hypothesis testing.

## The paper explained

### Problem

Hypoxia-inducible transcription factors (HIF) have long been involved in regulating tumor progression in a variety of solid tumors. More recently, emerging literature is beginning to implicate HIF factors in leukemogenesis. In acute myeloid leukemia, HIF-1α has been found overexpressed in a sub-group of cancer cells with leukemia propagating capacity and was shown to regulate leukemia stem cell maintenance. However, a specific functional interaction of HIF-1α with oncogenic fusion proteins causative of specific leukemia sub-types has not yet been investigated.

### Results

We have studied the role of HIF-1α in acute promyelocytic leukemia (APL), a sub-type of acute myeloid leukemia characterized by expression of the fusion protein PML-RARα. We found that although acting predominantly as a transcriptional repressor, PML-RARα functionally cooperates with HIF-1α and activates the expression of a number of HIF-target genes, which then regulate leukemia progression at multiple levels. As a consequence, inhibition of HIF-1α blunts leukemia progression, and also exquisitely cooperates with retinoic acid in eradicating leukemia-initiating cells.

### Impact

The impact of our study is twofold: on one hand, we provide molecular insights into the complex function of the PML-RARα oncoprotein, by defining a new important function of PML-RARα as a HIF-1α-transcriptional co-activator. On the other hand, our work places HIF factors as important regulators of the pathogenesis and response to therapy of acute promyelocytic leukemia, and prompt further investigation into the role of HIF-1α factors in other types of leukemia.

## For more information

http://www.sanraffaele.org/preclinical_models_of_cancer.html

## References

[b1] Ablain J, de The H (2011). Revisiting the differentiation paradigm in acute promyelocytic leukemia. Blood.

[b2] Bernardi R, Guernah I, Jin D, Grisendi S, Alimonti A, Teruya-Feldstein J, Cordon-Cardo C, Simon MC, Rafii S, Pandolfi PP (2006). PML inhibits HIF-1 alpha translation and neoangiogenesis through repression of mTOR. Nature.

[b3] Bernardi R, Scaglioni PP, Bergmann S, Horn HF, Vousden KH, Pandolfi PP (2004). PML regulates p53 stability by sequestering Mdm2 to the nucleolus. Nat Cell Biol.

[b4] Daniel MT, Koken M, Romagne O, Barbey S, Bazarbachi A, Stadler M, Guillemin MC, Degos L, Chomienne C, de The H (1993). PML protein expression in hematopoietic and acute promyelocytic leukemia cells. Blood.

[b5] Doucas V, Brockes JP, Yaniv M, de The H, Dejean A (1993). The PML-retinoic acid receptor alpha translocation converts the receptor from an inhibitor to a retinoic acid-dependent activator of transcription factor AP-1. Proc Natl Acad Sci USA.

[b6] Follenzi A, Ailles LE, Bakovic S, Geuna M, Naldini L (2000). Gene transfer by lentiviral vectors is limited by nuclear translocation and rescued by HIV-1 pol sequences. Nat Genet.

[b7] Greenberger LM, Horak ID, Filpula D, Sapra P, Westergaard M, Frydenlund HF, Albaek C, Schroder H, Orum H (2008). A RNA antagonist of hypoxia-inducible factor-1 alpha, EZN-2968, inhibits tumor cell growth. Mol Cancer Ther.

[b8] Grignani F, De Matteis S, Nervi C, Tomassoni L, Gelmetti V, Cioce M, Fanelli M, Ruthardt M, Ferrara FF, Zamir I (1998). Fusion proteins of the retinoic acid receptor-alpha recruit histone deacetylase in promyelocytic leukaemia. Nature.

[b9] Gutierrez NC, Lopez-Perez R, Hernandez JM, Isidro I, Gonzalez B, Delgado M, Ferminan E, Garcia JL, Vazquez L, Gonzalez M (2005). Gene expression profile reveals deregulation of genes with relevant functions in the different subclasses of acute myeloid leukemia. Leukemia.

[b10] Kini AR, Peterson LA, Tallman MS, Lingen MW (2001). Angiogenesis in acute promyelocytic leukemia: induction by vascular endothelial growth factor and inhibition by all-trans retinoic acid. Blood.

[b11] Lee KE, Simon MC (2012). From stem cells to cancer stem cells: HIF takes the stage. Curr Opin Cell Biol.

[b12] Lin TL, Wang QH, Brown P, Peacock C, Merchant AA, Brennan S, Jones E, McGovern K, Watkins DN, Sakamoto KM (2010). Self-renewal of acute lymphocytic leukemia cells is limited by the Hedgehog pathway inhibitors cyclopamine and IPI-926. PLoS ONE.

[b13] Lo-Coco F, Avvisati G, Vignetti M, Breccia M, Gallo E, Rambaldi A, Paoloni F, Fioritoni G, Ferrara F, Specchia G (2010). Front-line treatment of acute promyelocytic leukemia with AIDA induction followed by risk-adapted consolidation for adults younger than 61 years: results of the AIDA-2000 trial of the GIMEMA Group. Blood.

[b14] Lu X, Kang Y (2010). Hypoxia and hypoxia-inducible factors: master regulators of metastasis. Clin Cancer Res.

[b15] Marstrand TT, Borup R, Willer A, Borregaard N, Sandelin A, Porse BT, Theilgaard-Monch K (2010). A conceptual framework for the identification of candidate drugs and drug targets in acute promyelocytic leukemia. Leukemia.

[b16] Martens JH, Brinkman AB, Simmer F, Francoijs KJ, Nebbioso A, Ferrara F, Altucci L, Stunnenberg HG (2010). PML-RARalpha/RXR Alters the Epigenetic Landscape in Acute Promyelocytic Leukemia. Cancer Cell.

[b17] Meani N, Minardi S, Licciulli S, Gelmetti V, Coco FL, Nervi C, Pelicci PG, Muller H, Alcalay M (2005). Molecular signature of retinoic acid treatment in acute promyelocytic leukemia. Oncogene.

[b18] Minucci S, Monestiroli S, Giavara S, Ronzoni S, Marchesi F, Insinga A, Diverio D, Gasparini P, Capillo M, Colombo E (2002). PML-RAR induces promyelocytic leukemias with high efficiency following retroviral gene transfer into purified murine hematopoietic progenitors. Blood.

[b19] Nasr R, Guillemin MC, Ferhi O, Soilihi H, Peres L, Berthier C, Rousselot P, Robledo-Sarmiento M, Lallemand-Breitenbach V, Gourmel B (2008). Eradication of acute promyelocytic leukemia-initiating cells through PML-RARA degradation. Nat Med.

[b20] Nombela-Arrieta C, Pivarnik G, Winkel B, Canty KJ, Harley B, Mahoney JE, Park SY, Lu J, Protopopov A, Silberstein LE (2013). Quantitative imaging of haematopoietic stem and progenitor cell localization and hypoxic status in the bone marrow microenvironment. Nat Cell Biol.

[b21] Rouault-Pierre K, Lopez-Onieva L, Foster K, Anjos-Afonso F, Lamrissi-Garcia I, Serrano-Sanchez M, Mitter R, Ivanovic Z, de Verneuil H, Gribben J (2013). HIF-2alpha Protects Human Hematopoietic Stem/Progenitors and Acute Myeloid Leukemic Cells from Apoptosis Induced by Endoplasmic Reticulum Stress. Cell Stem Cell.

[b22] Sanz MA, Montesinos P, Vellenga E, Rayon C, de la Serna J, Parody R, Bergua JM, Leon A, Negri S, Gonzalez M (2008). Risk-adapted treatment of acute promyelocytic leukemia with all-trans retinoic acid and anthracycline monochemotherapy: long-term outcome of the LPA 99 multicenter study by the PETHEMA Group. Blood.

[b23] Subramanian A, Tamayo P, Mootha VK, Mukherjee S, Ebert BL, Gillette MA, Paulovich A, Pomeroy SL, Golub TR, Lander ES (2005). Gene set enrichment analysis: a knowledge-based approach for interpreting genome-wide expression profiles. Proc Natl Acad Sci USA.

[b24] Takubo K, Goda N, Yamada W, Iriuchishima H, Ikeda E, Kubota Y, Shima H, Johnson RS, Hirao A, Suematsu M (2010). Regulation of the HIF-1alpha level is essential for hematopoietic stem cells. Cell Stem Cell.

[b25] Tavor S, Petit I, Porozov S, Avigdor A, Dar A, Leider-Trejo L, Shemtov N, Deutsch V, Naparstek E, Nagler A (2004). CXCR4 regulates migration and development of human acute myelogenous leukemia stem cells in transplanted NOD/SCID mice. Cancer Res.

[b26] Theilgaard-Monch K, Jacobsen LC, Borup R, Rasmussen T, Bjerregaard MD, Nielsen FC, Cowland JB, Borregaard N (2005). The transcriptional program of terminal granulocytic differentiation. Blood.

[b27] Tsuzuki S, Towatari M, Saito H, Enver T (2000). Potentiation of GATA-2 activity through interactions with the promyelocytic leukemia protein (PML) and the t(15;17)-generated PML-retinoic acid receptor alpha oncoprotein. Mol Cell Biol.

[b28] Valk PJ, Verhaak RG, Beijen MA, Erpelinck CA, Barjesteh van Waalwijk van Doorn-Khosrovani S, Boer JM, Beverloo HB, Moorhouse MJ, van der Spek PJ, Lowenberg B (2004). Prognostically useful gene-expression profiles in acute myeloid leukemia. N Engl J Med.

[b29] Viale A, De Franco F, Orleth A, Cambiaghi V, Giuliani V, Bossi D, Ronchini C, Ronzoni S, Muradore I, Monestiroli S (2009). Cell-cycle restriction limits DNA damage and maintains self-renewal of leukaemia stem cells. Nature.

[b30] Wang K, Wang P, Shi J, Zhu X, He M, Jia X, Yang X, Qiu F, Jin W, Qian M (2010). PML/RARalpha targets promoter regions containing PU.1 consensus and RARE half sites in acute promyelocytic leukemia. Cancer Cell.

[b31] Wang Y, Liu Y, Malek SN, Zheng P (2011). Targeting HIF1alpha eliminates cancer stem cells in hematological malignancies. Cell Stem Cell.

[b32] Wenger RH, Stiehl DP, Camenisch G (2005). Integration of oxygen signaling at the consensus HRE. Sci STKE.

[b33] Wu Z, Irizarry RA (2004). Preprocessing of oligonucleotide array data. Nat Biotechnol.

[b34] Zhang H, Li H, Xi HS, Li S (2012). HIF1alpha is required for survival maintenance of chronic myeloid leukemia stem cells. Blood.

[b35] Zheng X, Seshire A, Ruster B, Bug G, Beissert T, Puccetti E, Hoelzer D, Henschler R, Ruthardt M (2007). Arsenic but not all-trans retinoic acid overcomes the aberrant stem cell capacity of PML/RARalpha-positive leukemic stem cells. Haematologica.

