# Supplementary material for: HIF factors cooperate with PML-RARα to promote acute promyelocytic leukemia progression and relapse
Source: EMBO Mol Med. 2014 Apr 7;6(5):640–50. doi: 10.1002/emmm.201303065 (PMC4023886; doi:10.1002/emmm.201303065)
Supplement: Supplementary file 6 [file emmm0006-0640-sd6.pdf]

**Supporting Information Figure 1 Panel A
(Left Panel, upper part)**

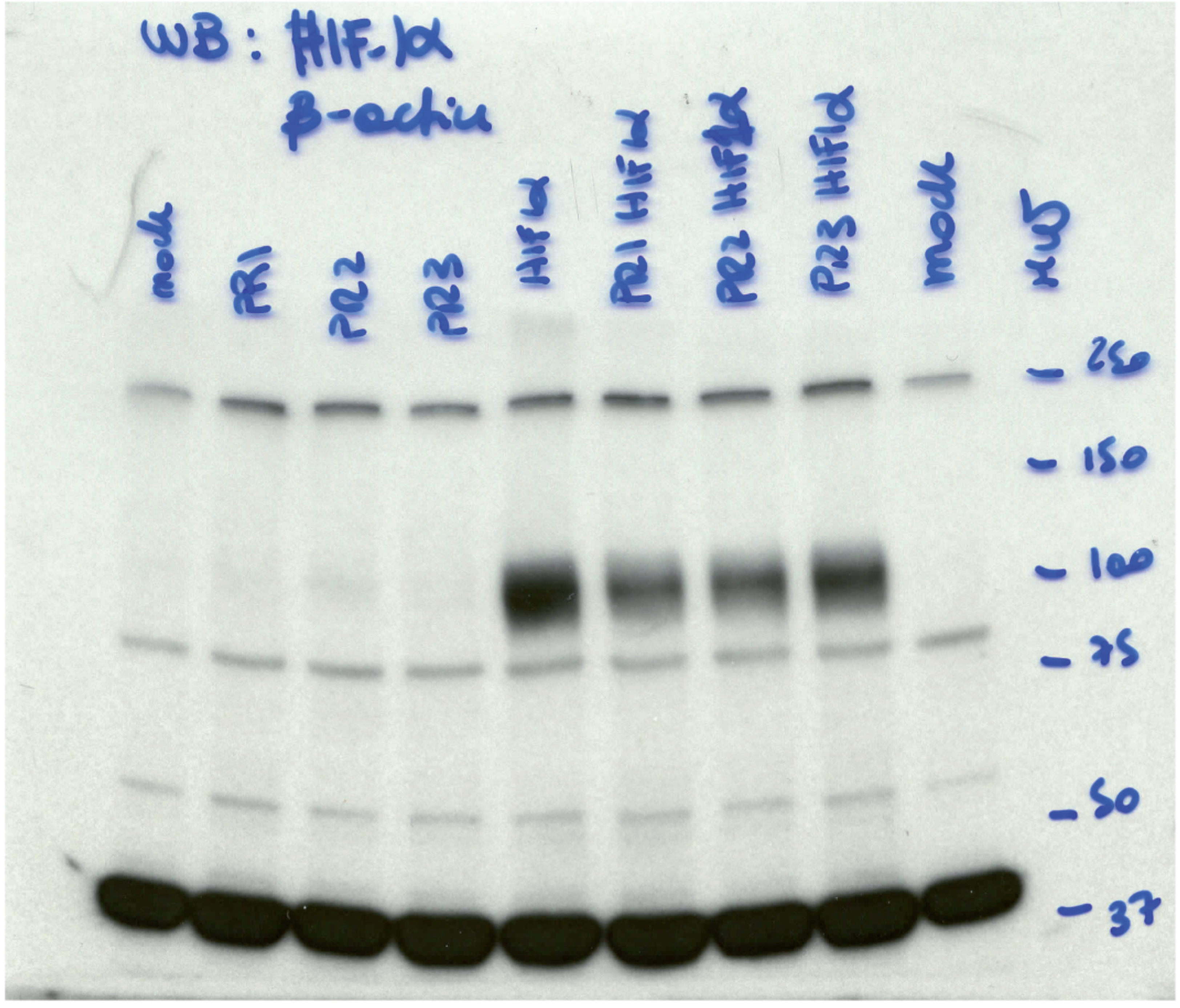

WB: anti-HIF-1α + anti-β-actin

**Supporting Information Figure 1 Panel A
(Left Panel, lower part)**

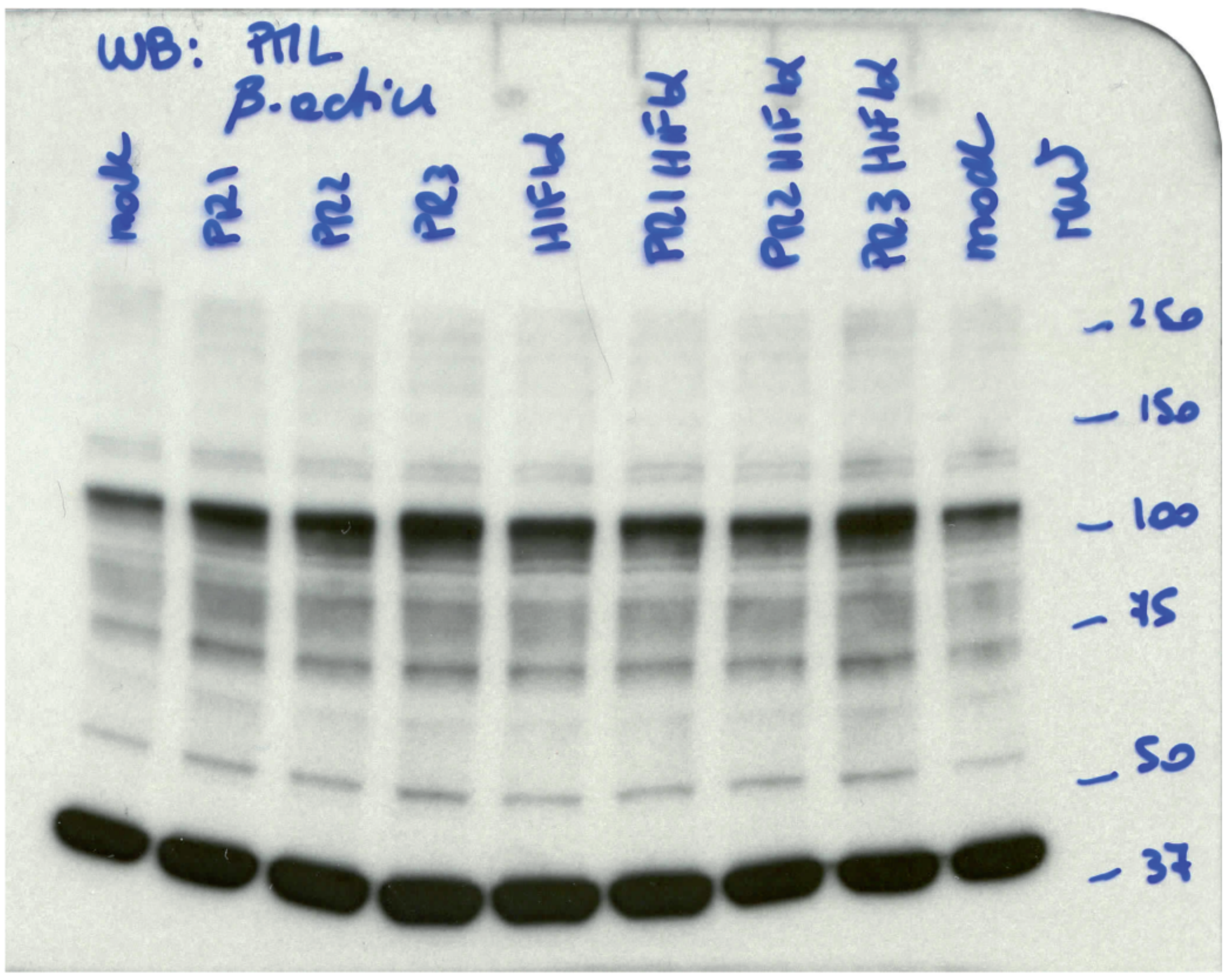

WB: anti-PML + anti-β-actin

**Supporting Information Figure 1 Panel A
(Right Panel, upper part)**

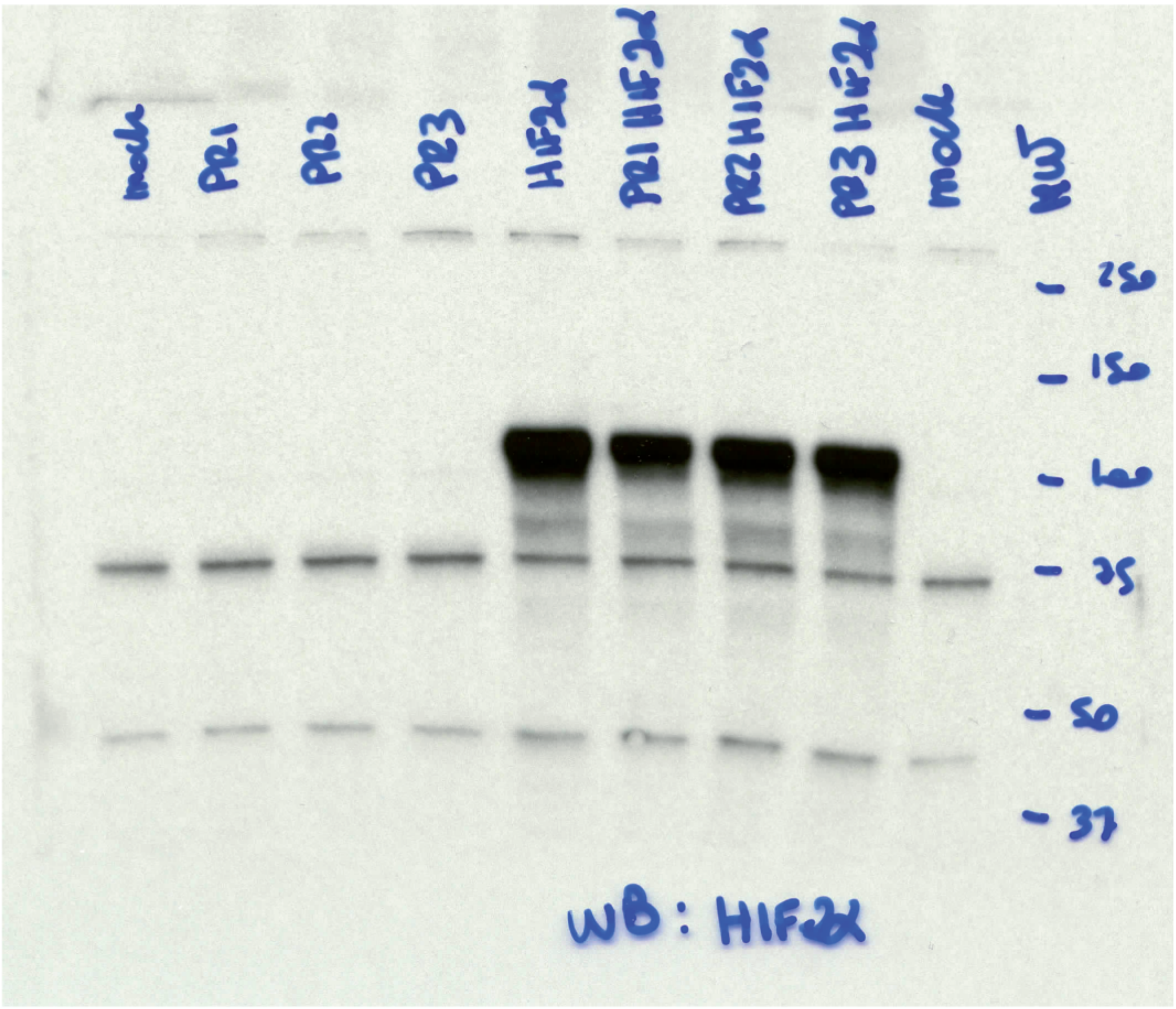

WB: anti-HIF-2α

**Supporting Information Figure 1 Panel A
(Right Panel, lower part)**

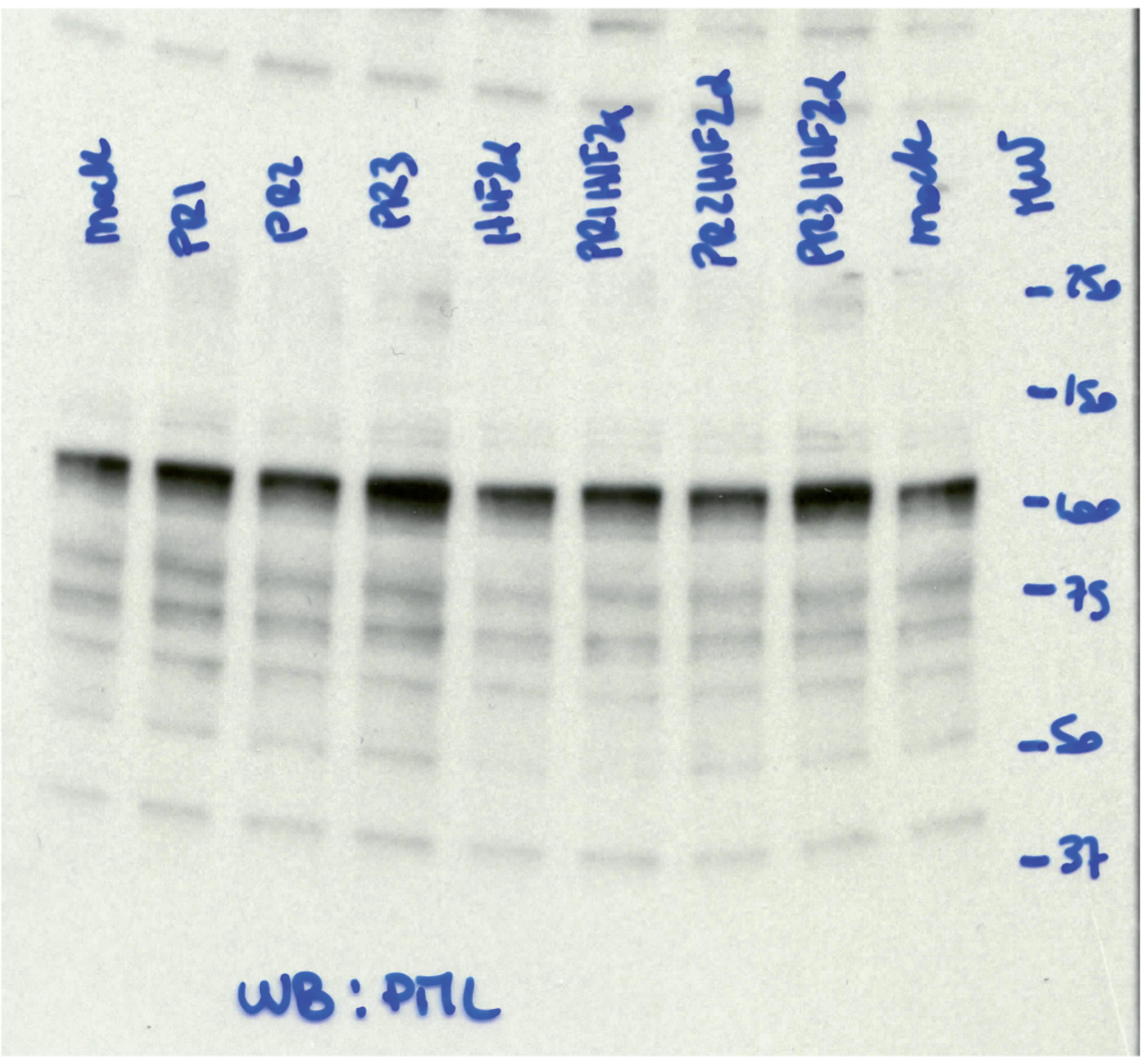

WB: anti-PML

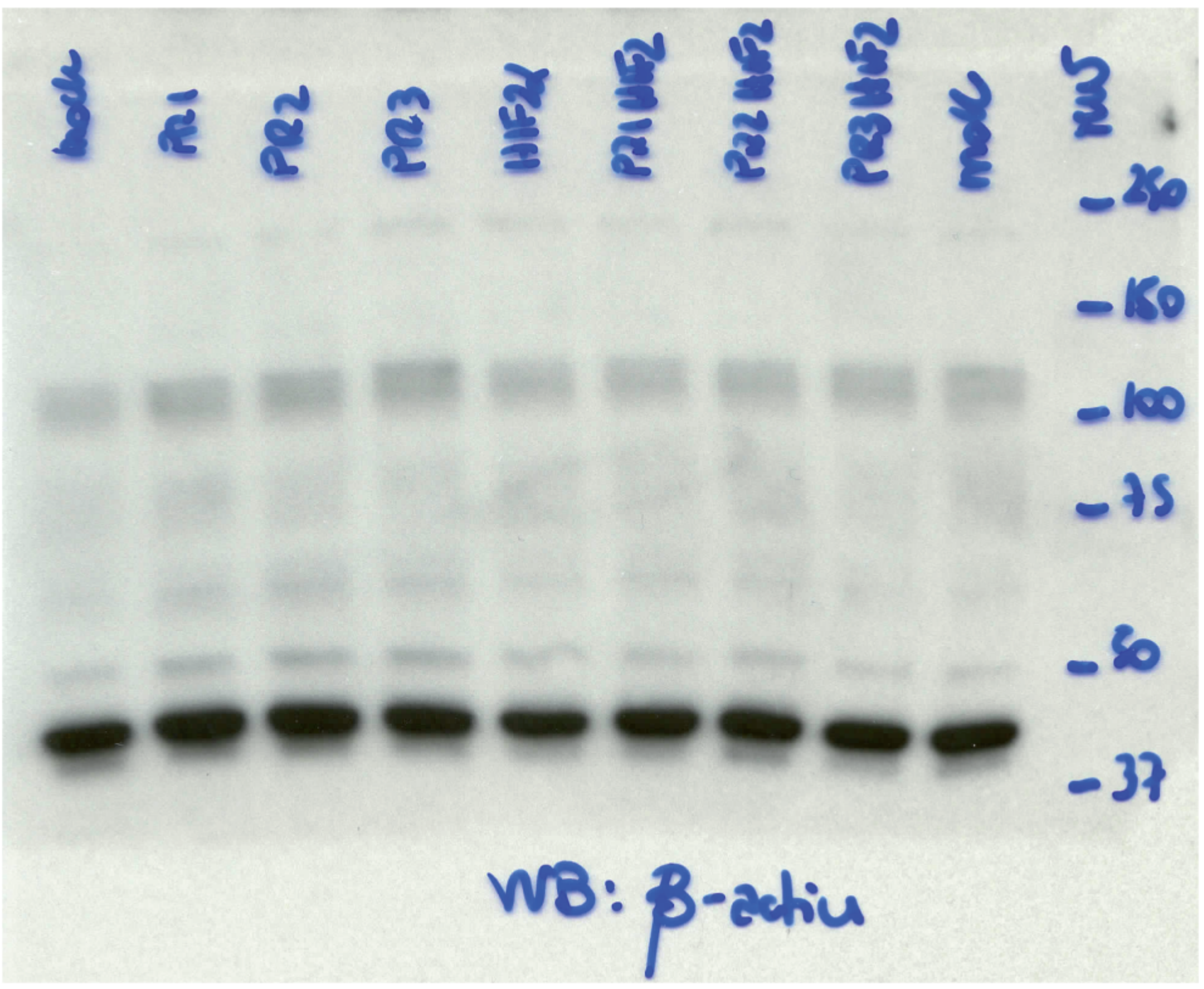

WB: anti-β-actin (after stripping)

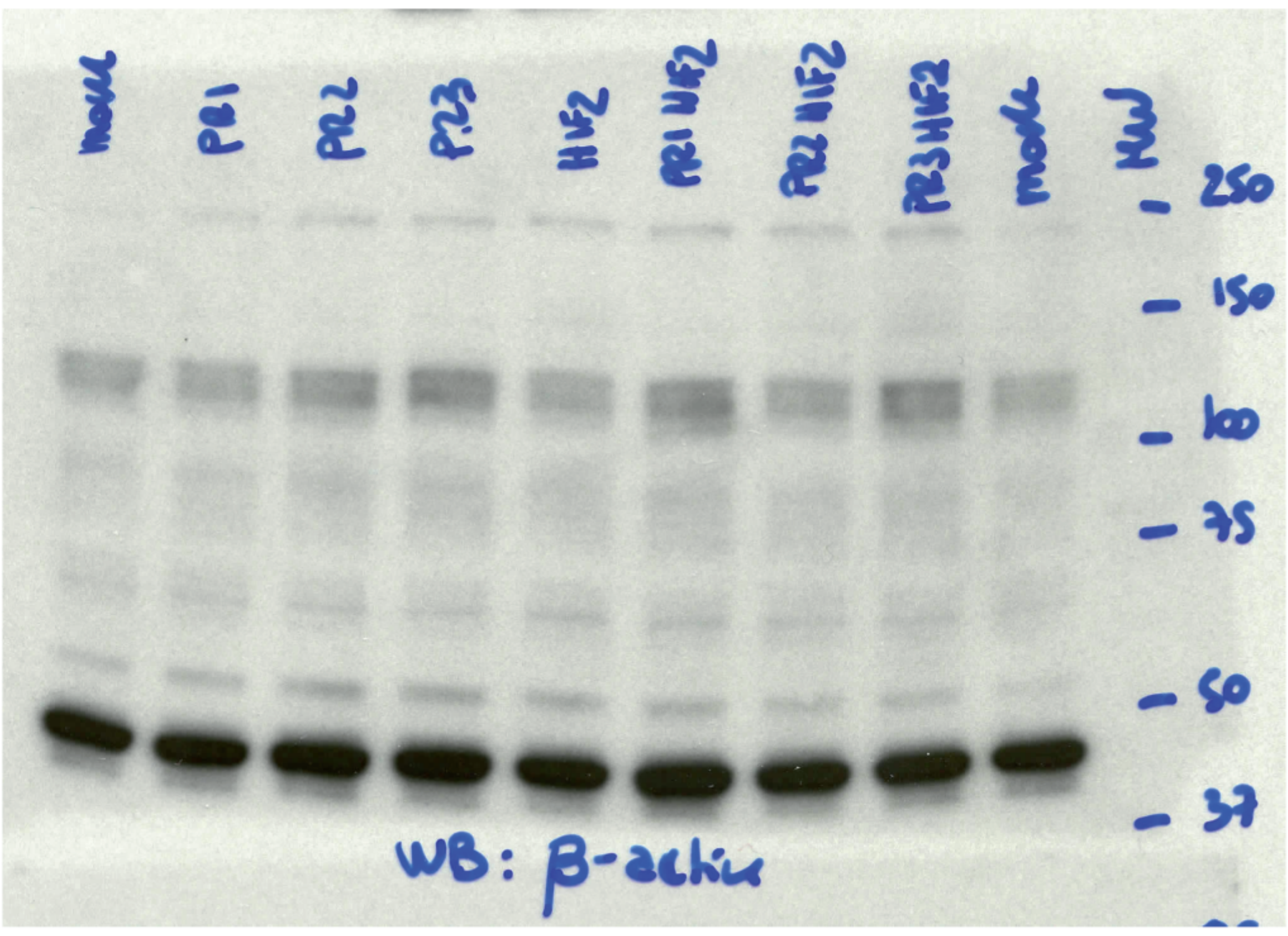

WB: anti-β-actin (after stripping)
