# Supplementary material for: HIF factors cooperate with PML-RARα to promote acute promyelocytic leukemia progression and relapse
Source: EMBO Mol Med. 2014 Apr 7;6(5):640–50. doi: 10.1002/emmm.201303065 (PMC4023886; doi:10.1002/emmm.201303065)
Supplement: Supplementary file 7 [file emmm0006-0640-sd7.pdf]

EMBO Molecular Medicine

HIF factors cooperate with PML-RARα to promote acute promyelocytic leukemia progression and relapse

Nadia Coltella, Stefano Percio, Roberta Valsecchi, Roberto Cuttano, Jlenia Guarnerio, Maurilio Ponzoni, Pier Paolo Pandolfi, Giovanni Melillo, Linda Pattini and Rosa Bernardi

Corresponding author: Rosa Bernardi, San Raffaele Scientific Institute

Review timeline:

Submission date:	21 May 2013
Accepted:	28 February 2014

Editor: Roberto Buccione

Transaction Report:

No Peer Review Process File is available with this article, as the authors have chosen not to make the review process public in this case.

© EMBO
