# Supplementary material for: HIF factors cooperate with PML-RARα to promote acute promyelocytic leukemia progression and relapse
Source: EMBO Mol Med. 2014 Apr 7;6(5):640–50. doi: 10.1002/emmm.201303065 (PMC4023886; doi:10.1002/emmm.201303065)
Supplement: Supplementary file 8 [file emmm0006-0640-sd8.pdf]

Figure 1 Panel E (Left Upper Panel)

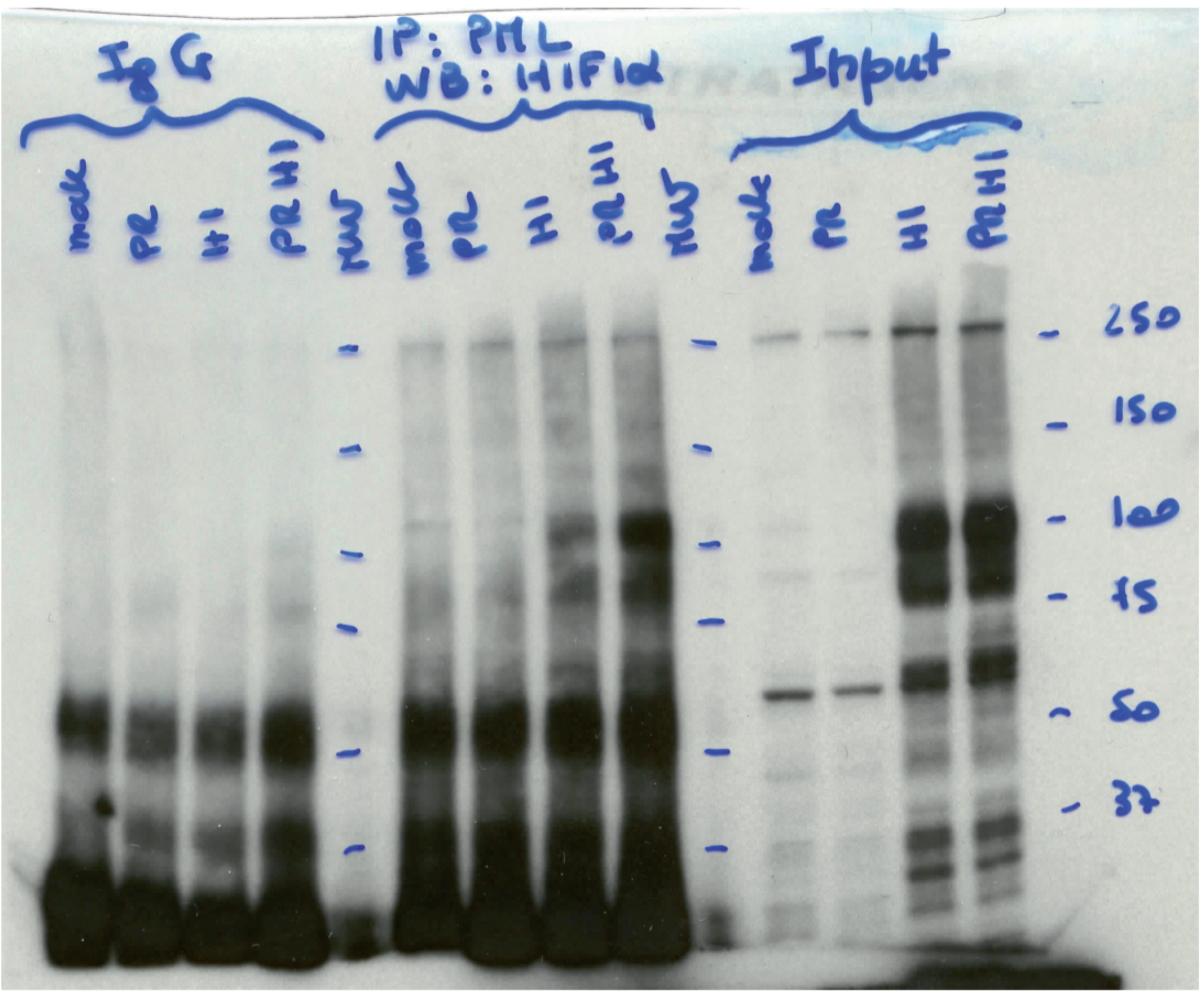

Figure 1 Panel E (Left Lower Panel)

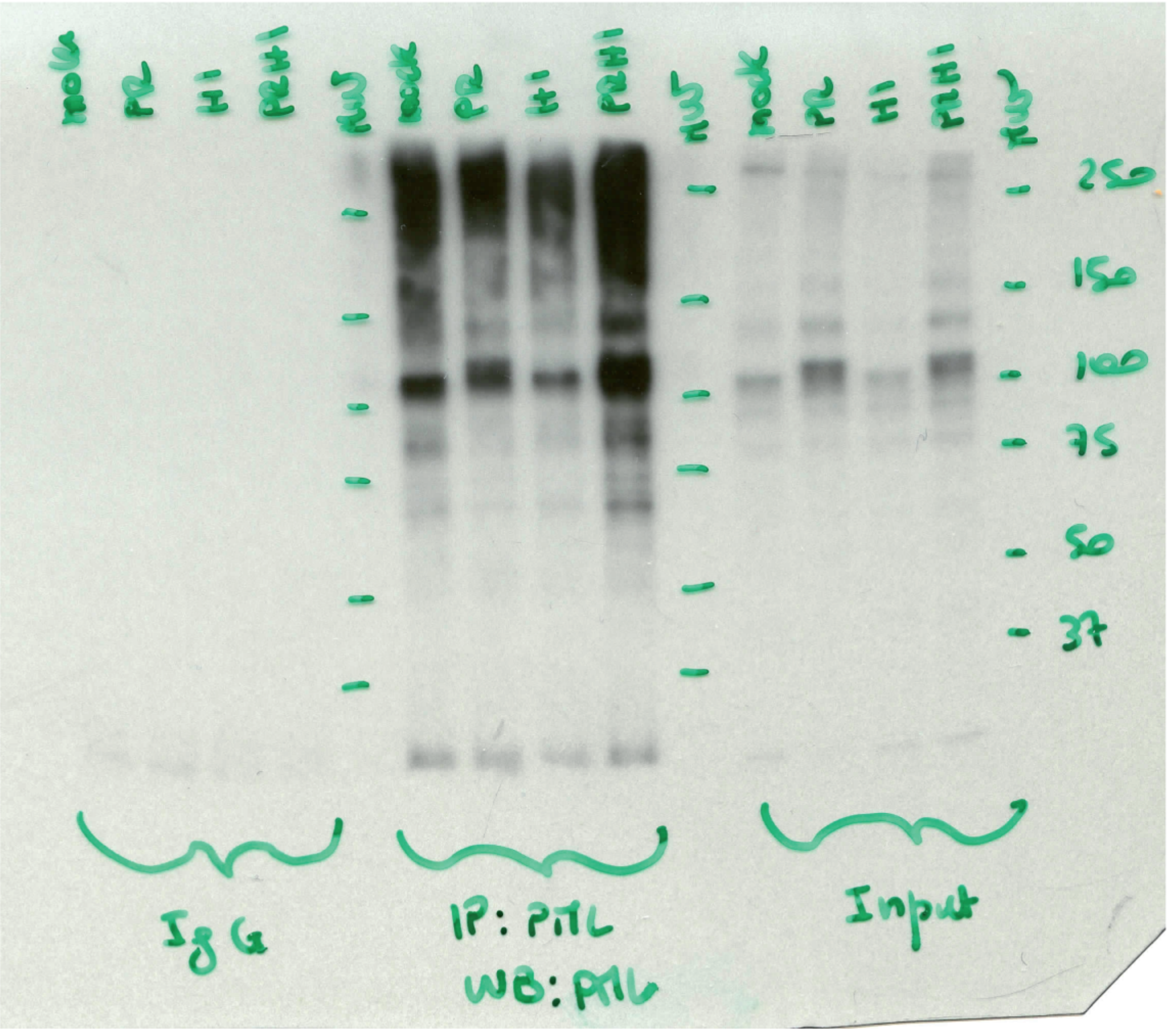

Figure 1 Panel E (Right Upper Panel)

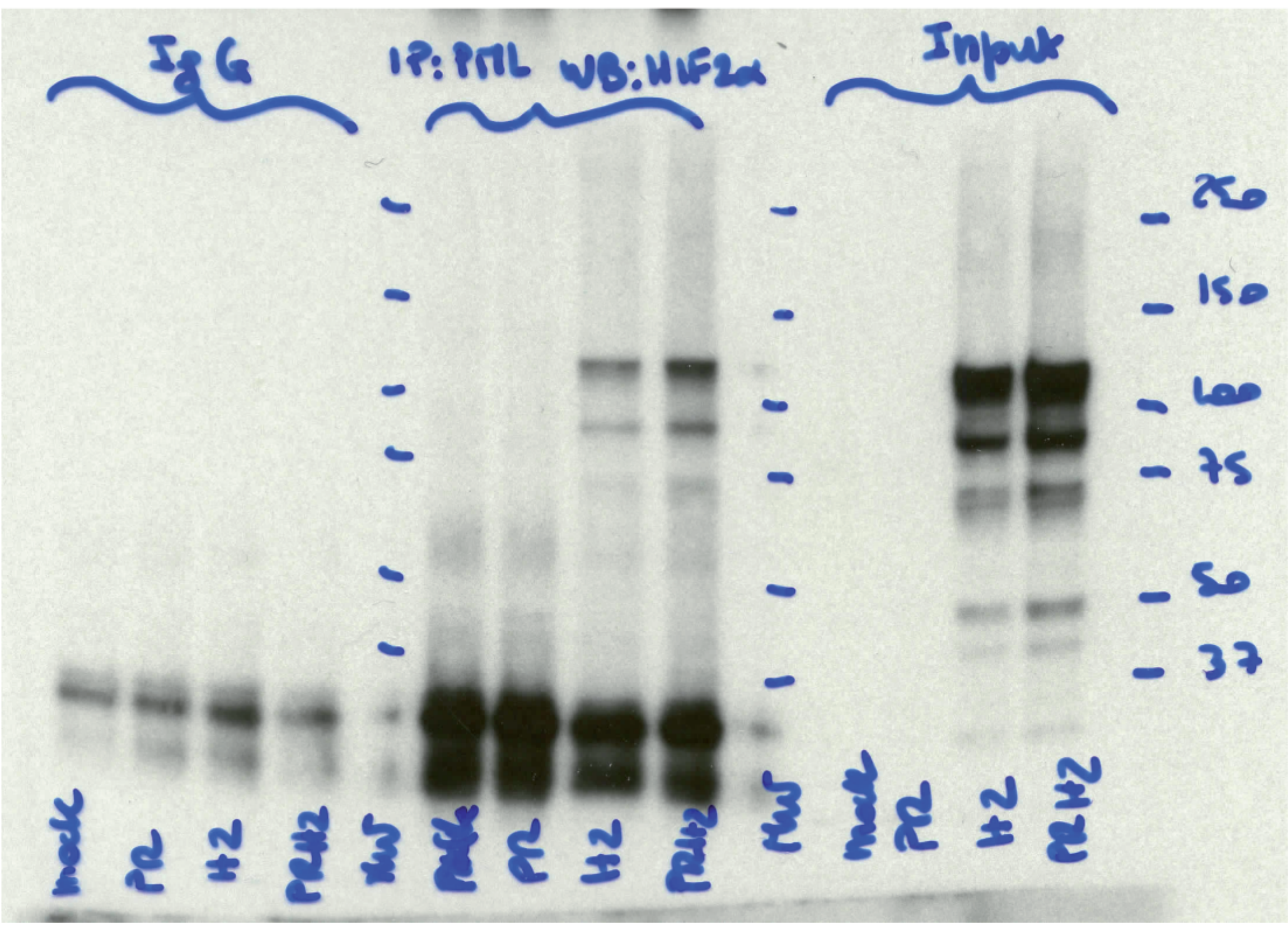

Figure 1 Panel E (Right Lower Panel)

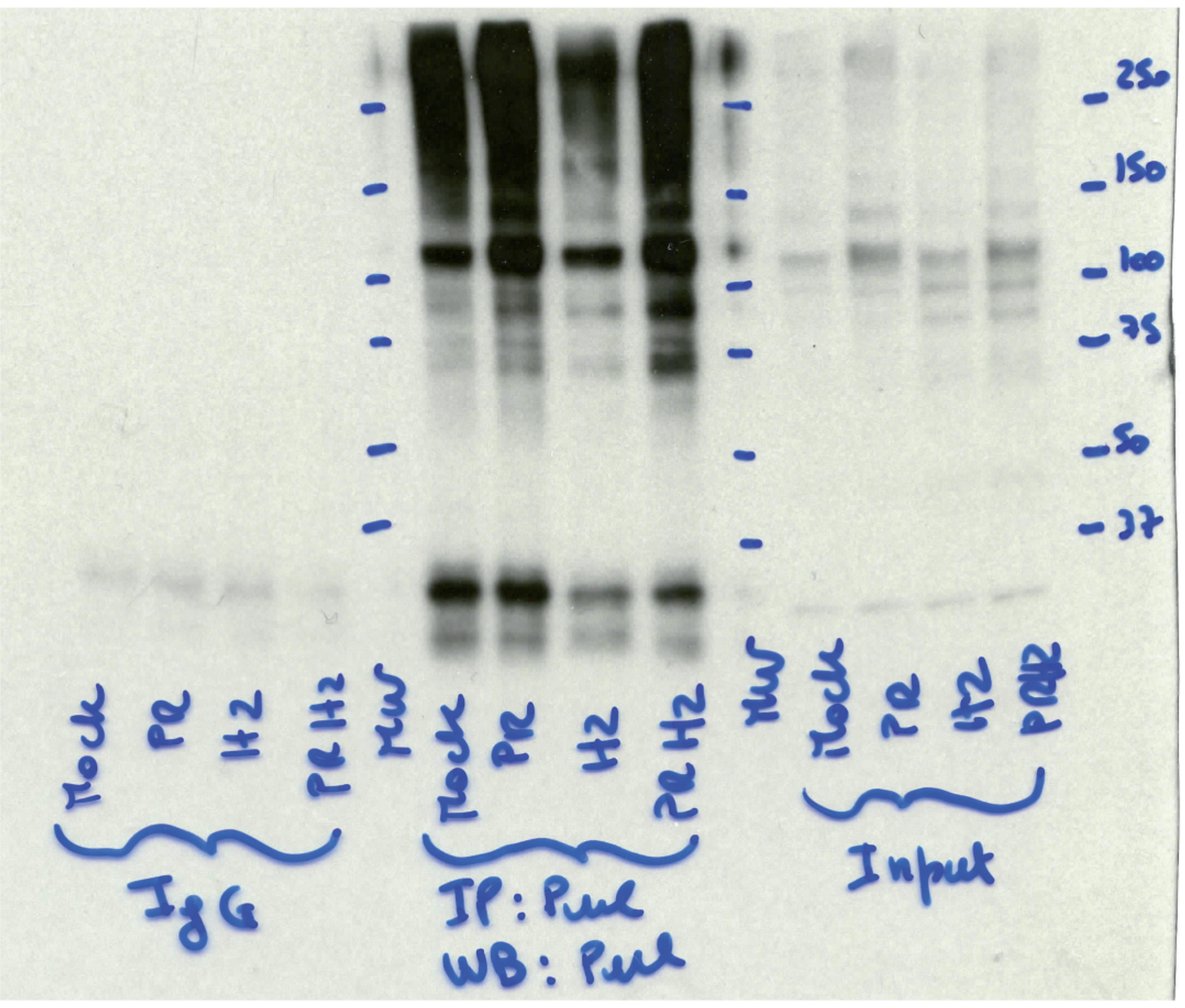

Figure 1 Panel F (Left Panel)

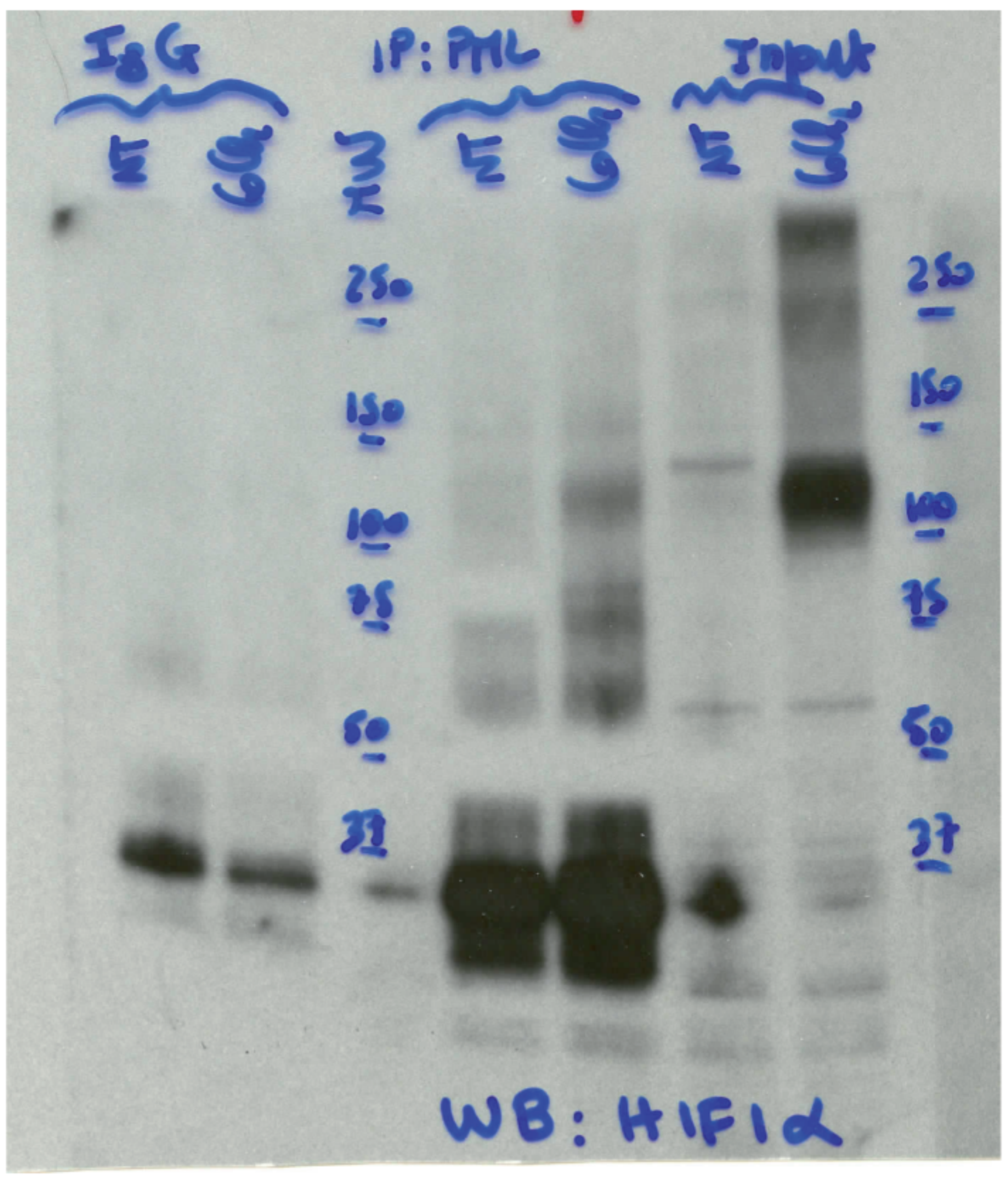

IP: PML
WB: anti-HIF-1α

Figure 1 Panel F (Left Panel) higher exposure time

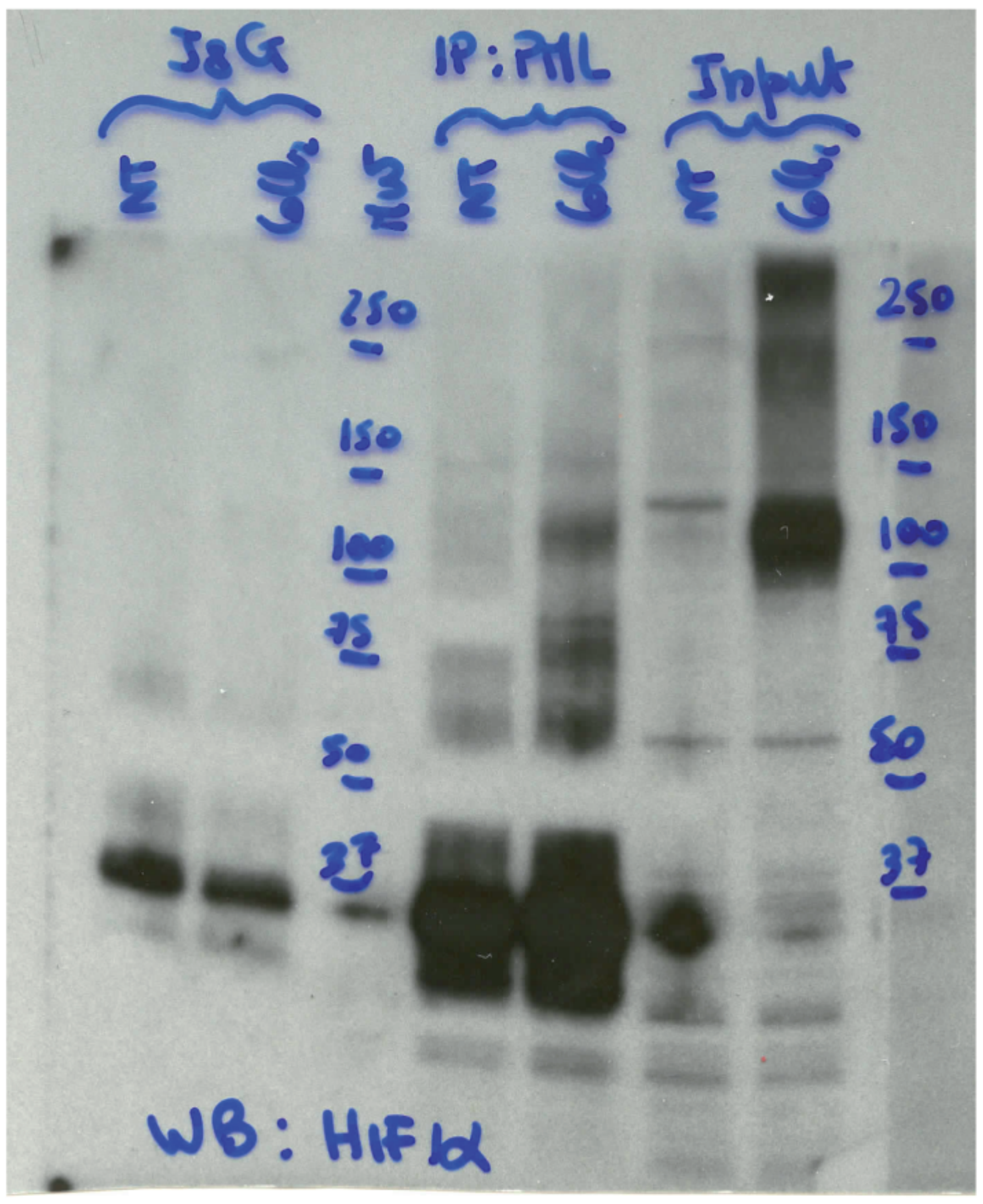

IP: PML
WB: anti-HIF-1α

Figure 1 Panel F (Right Panel)

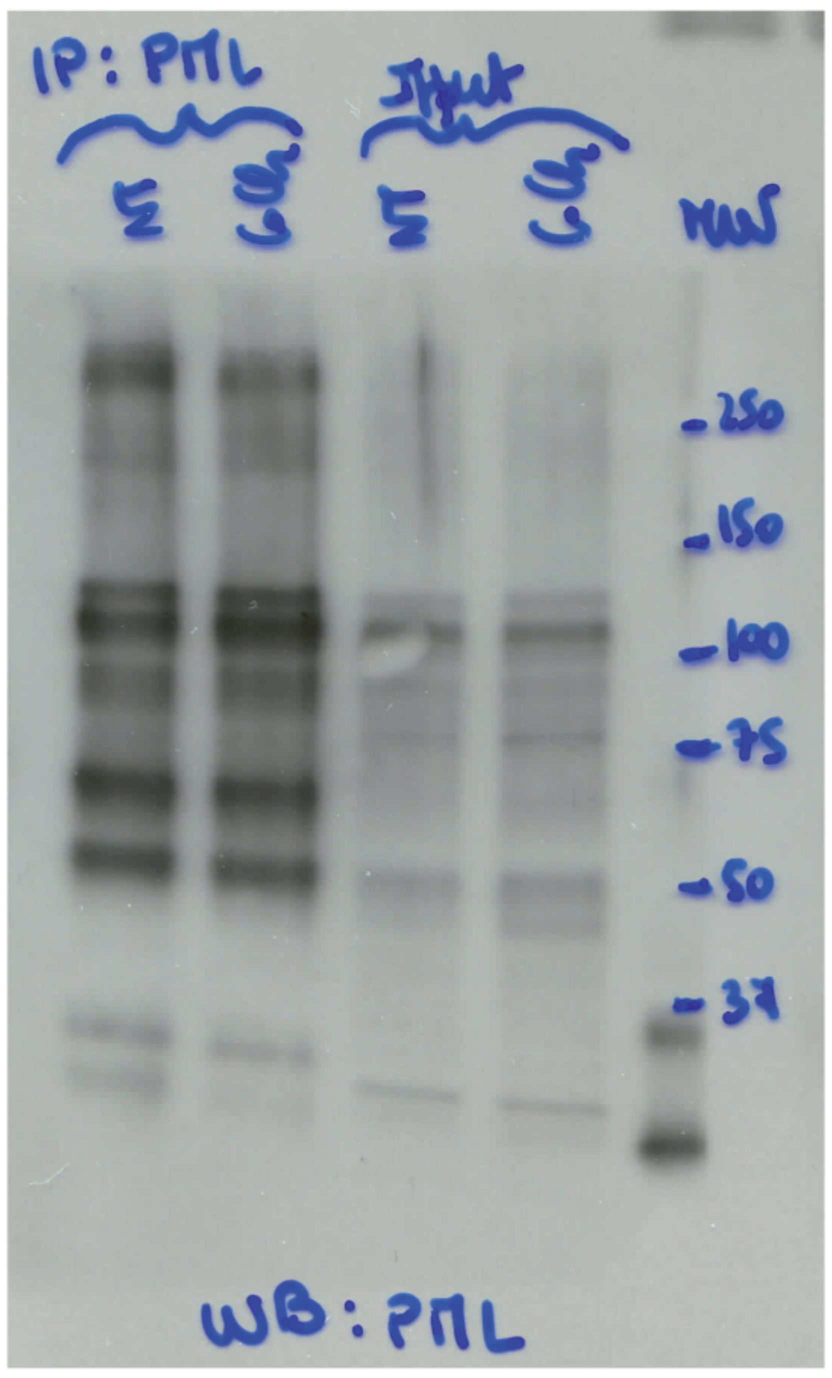

IP: PML
WB: anti-PML
