# Supplementary material for: HIF factors cooperate with PML-RARα to promote acute promyelocytic leukemia progression and relapse
Source: EMBO Mol Med. 2014 Apr 7;6(5):640–50. doi: 10.1002/emmm.201303065 (PMC4023886; doi:10.1002/emmm.201303065)
Supplement: Supplementary file 9 [file emmm0006-0640-sd9.pdf]

Figure 3 Panel B

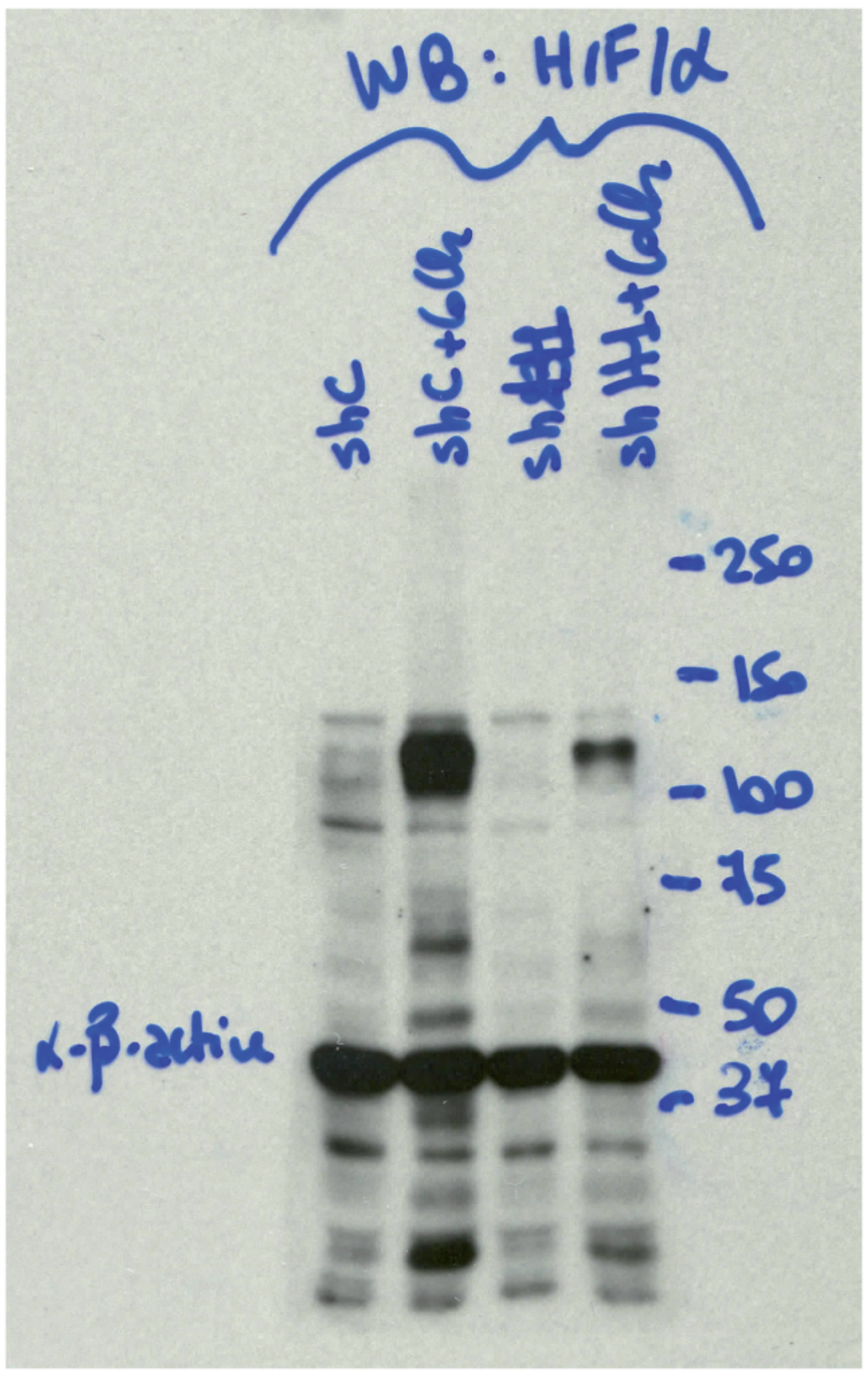

WB: anti-HIF-1α + anti-β-actin

Figure 3 Panel B
higher exposure time

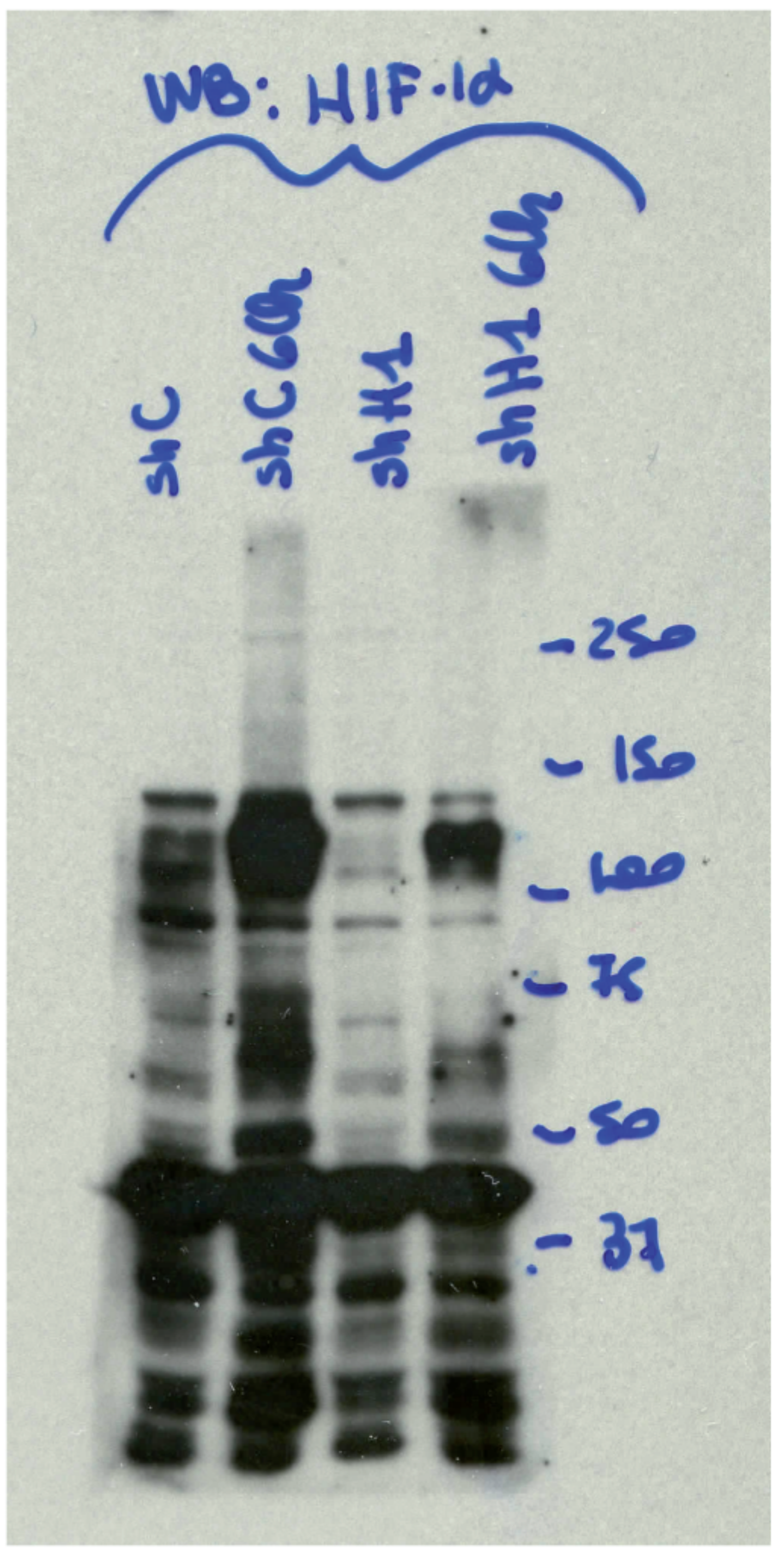

WB: anti-HIF-1α + anti-β-actin
