# Supplementary material for: HIF factors cooperate with PML-RARα to promote acute promyelocytic leukemia progression and relapse
Source: EMBO Mol Med. 2014 Apr 7;6(5):640–50. doi: 10.1002/emmm.201303065 (PMC4023886; doi:10.1002/emmm.201303065)
Supplement: Supplementary file 1 [file emmm0006-0640-sd1.pdf]

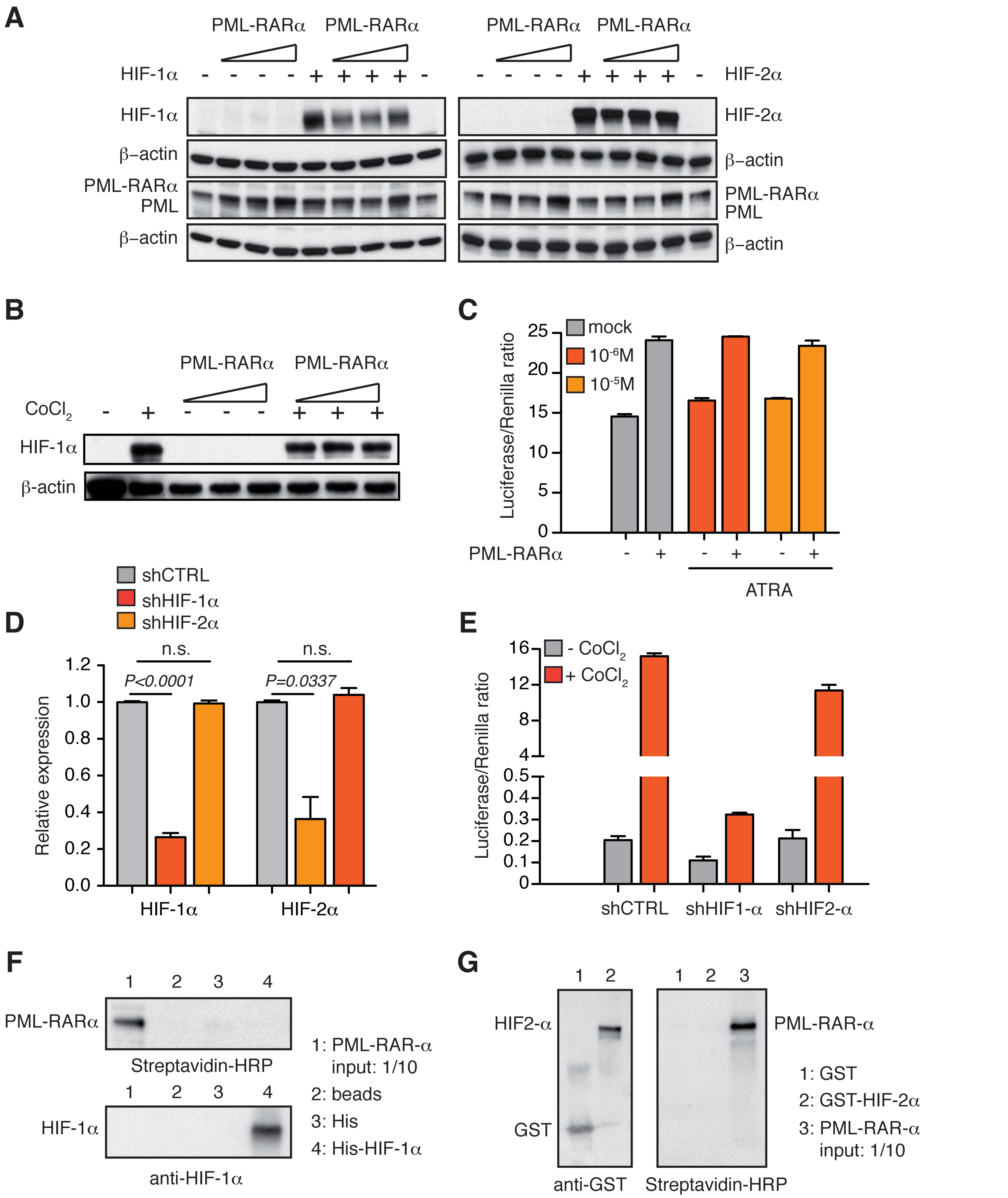

PML-RARα induced HIF-1α transcription is not dependent on protein accumulation, ATRA treatment or direct interaction. (A) Immunoblot analysis of HIF-1α (left panel), HIF-2α (righ panel) and PML-RARα in HEK-293 cells transfected with increasing concentrations of PML-RARα and stable forms of HIF-1α and HIF-2α. (B) Immunoblot analysis of HIF-1α expression in HEK-293 cells transfected with increasing concentrations of PML-RARα and treated with $CoCl_2$. (C) HEK-293 cells transfected with a stable form of HIF-1α and PML-RARα and treated with the indicated ATRA concentrations for 24 hrs. (D) Real-time PCR analysis of HIF-1α and HIF-2α expression in shCTRL, shHIF-1α and shHIF-2α HEK-293 cells. (E) HIF-1α transactivation assays with HRE-luciferase construct. HEK-293 cells (shCTRL, shHIF-1α and shHIF-2α) were treatead with $CoCl_2$. Results are presented as mean ± s.e.m. of experiments performed in triplicate. (F) *In vitro* interaction assay with His-Tagged-HIF-1α (His-HIF-1α) and *in vitro* translated biotin labeled PML-RARα. Streptavidin-HRP antibody was used to dected labeled protein. HIF-1α monoclonal antibody was used to dected immunoprecipitated protein. (G) *In vitro* interaction assays with GST-Tagged-HIF2α (GST-HIF-2α) and *in vitro* translated biotin labeled PML-RARα.Streptavidin-HRP antibody was used to dected labeled protein.GST monoclonal antibody was used to dected immunoprecipitated protein
