# Supplementary material for: HIF factors cooperate with PML-RARα to promote acute promyelocytic leukemia progression and relapse
Source: EMBO Mol Med. 2014 Apr 7;6(5):640–50. doi: 10.1002/emmm.201303065 (PMC4023886; doi:10.1002/emmm.201303065)
Supplement: Supplementary file 2 [file emmm0006-0640-sd2.pdf]

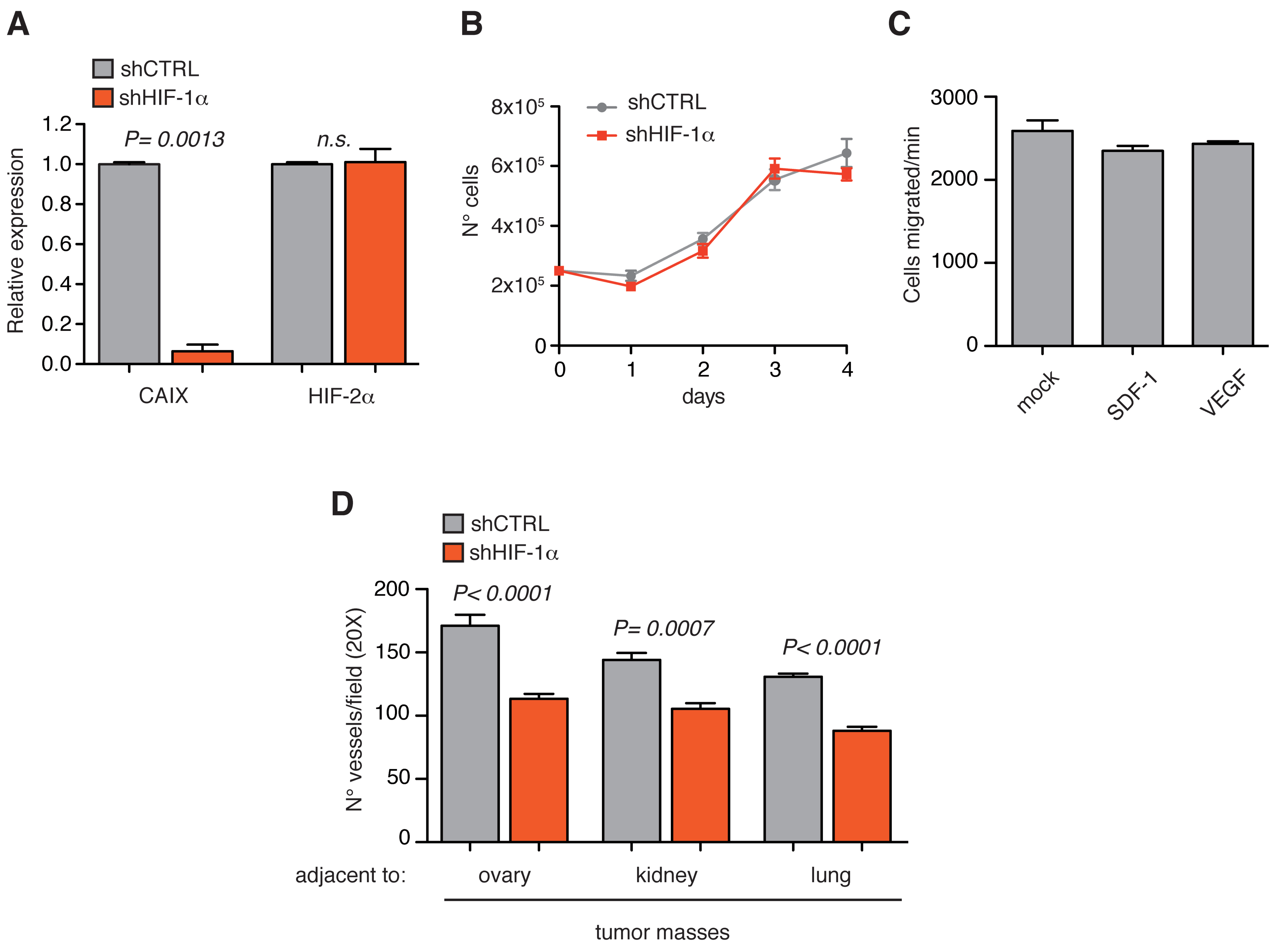

***In vitro* and *in vivo* characterization of NB4 cells after HIF-1α down-regulation.**
(A) Real-time PCR analysis of CA-IX and HIF-2α expression in shCTRL or shHIF-1α NB4 cells. Data represent mean values ± s.e.m. of two independent experiments. (B) Proliferation curve of shCTRL or shHIF-1α NB4 cells.
(C) Spontaneous, SDF-1 and VEGF induced migration of NB4 cells in transwell migration assays.
(D) CD31 immunostaining of tumor masses from mice challenged with shCTRL or shHIF-1α NB4 cells. Graphs show average number of microvessels per field (20X).
