# Supplementary material for: HIF factors cooperate with PML-RARα to promote acute promyelocytic leukemia progression and relapse
Source: EMBO Mol Med. 2014 Apr 7;6(5):640–50. doi: 10.1002/emmm.201303065 (PMC4023886; doi:10.1002/emmm.201303065)
Supplement: Supplementary file 3 [file emmm0006-0640-sd3.pdf]

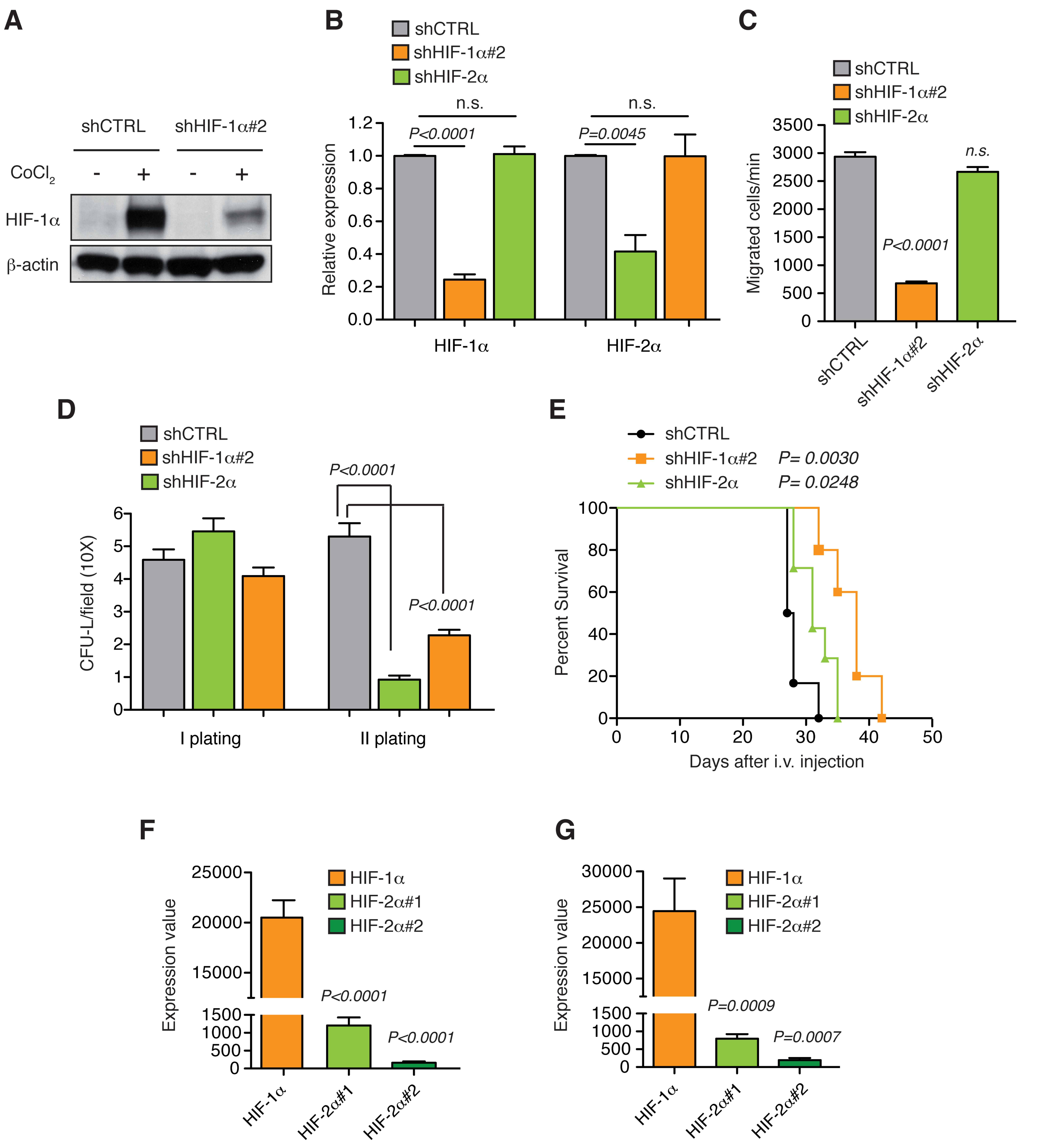

HIF-1α and HIF-2α inhibition impairs APL progression in a xenograft model.
(A) Immunoblot of HIF-1α in NB4 cells stably transduced with lentiviral vectors carrying control (shCTRL) or HIF-1α-directed shRNA (shHIF-1α#2) and treated with $CoCl_2$. (B) Real-time PCR analysis of HIF-1α and HIF-2α in shCTRL and shHIF-1α#2 or shHIF-2α NB4 cells. (C) Spontaneous migration of shCTRL, shHIF-1α#2 and shHIF-2α NB4 cells. Data are expressed as mean values ± s.e.m of triplicates from one representative experiment out of three with similar results. (D) Colony forming assay upon serial replating of shCTRL, shHIF-1α#2 and shHIF-2α NB4 cells. Data are expressed as mean values ± s.e.m of triplicates from one representative experiment out of two with similar results. (E) Kaplan-Meier survival curve of Rag2-/-γc-/- mice injected with shCTRL (n=6), shHIF-1α#2 (n=5) and shHIF-2α (n=7) NB4 cells. (F) mRNA expression of HIF-1α and HIF-2α in APL samples (n=14). (G) mRNA expression of HIF-1α and HIF-2α in CD34+ samples (n=5). The mean (and s.e.m.) expression values are reported for the probesets available for HIF-1α and HIF-2α (EPAS1) on the Affymetrix HG-U133 Plus 2.0 Array. All experiments were repeated at least twice.
