# Supplementary material for: HIF factors cooperate with PML-RARα to promote acute promyelocytic leukemia progression and relapse
Source: EMBO Mol Med. 2014 Apr 7;6(5):640–50. doi: 10.1002/emmm.201303065 (PMC4023886; doi:10.1002/emmm.201303065)
Supplement: Supplementary file 4 [file emmm0006-0640-sd4.pdf]

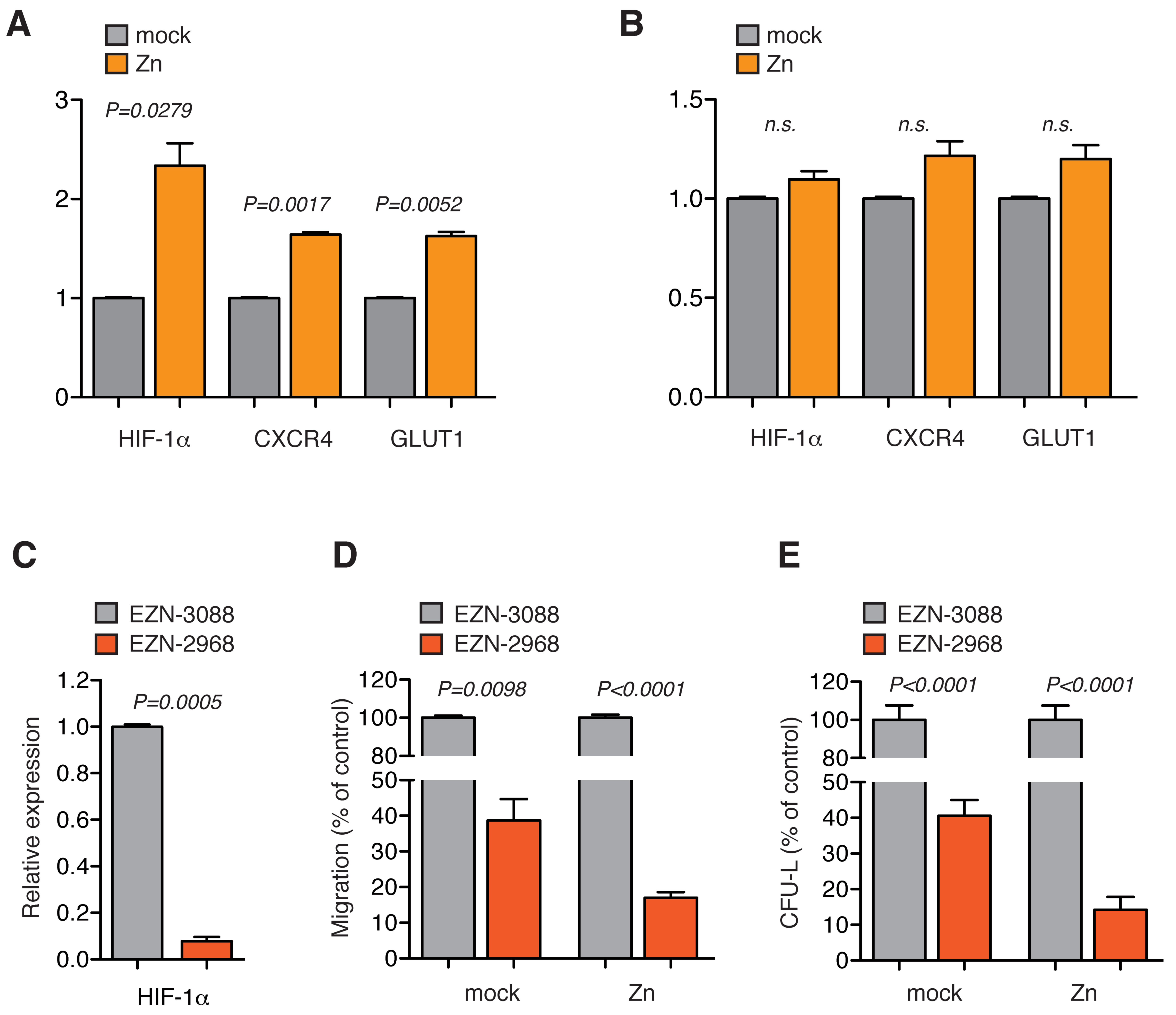

HIF-1α inhibition impairs cell migration and colony formation expecially upon expression of PML-RARα.
(A) Real-time PCR analysis of HIF-1α, CXCR4 and GLUT1 in U937-PR9 cells treated for 16 hrs with Zn. (B) Real-time PCR analysis of HIF-1α, CXCR4 and GLUT1 in U937-MT bulk cells treated for 16 hrs with Zn. (C) Real-time PCR analysis of HIF-1α in EZN-3088 or EZN-2968 transfected U937-PR9 cells 24 hrs after electroporation. (D) Spontaneous migration of U937-PR9 transfected with EZN-3088 or EZN-2968 and treated with Zn as indicated. Data are expressed as mean values ± s.e.m of triplicates from one representative experiment out of two with similar results. (E) Colony forming assay of U937-PR9 transfected with EZN-3088 or EZN-2968 and treated with Zn as indicated. All data are expressed as mean values ± s.e.m of triplicates from one representative experiment out of at least two with similar results.All experiments were repeated at least twice.
